# Estimated Exposure to 6 Potentially Hepatotoxic Botanicals in US Adults

**DOI:** 10.1001/jamanetworkopen.2024.25822

**Published:** 2024-08-05

**Authors:** Alisa Likhitsup, Vincent L. Chen, Robert J. Fontana

**Affiliations:** 1 Department of Internal Medicine, Division of Gastroenterology and Hepatology, University of Michigan, Ann Arbor, Michigan

## Abstract

**Importance:**

Use of herbal and dietary supplements (HDSs) accounts for an increasing proportion of drug hepatotoxicity cases. Turmeric or curcumin, green tea extract, *Garcinia cambogia*, black cohosh, red yeast rice, and ashwagandha are the most frequently reported hepatoxic botanicals, but their prevalence and reasons for use in the general population are unknown.

**Objective:**

To assess the prevalence and clinical characteristics of adult consumers of 6 potentially hepatoxic botanicals.

**Design, Setting, and Participants:**

This survey study analyzed nationally representative data from the National Health and Nutrition Examination Survey (NHANES), a nationally representative, cross-sectional survey of the general US population. Prescription drug and HDS exposure data in the past 30 days were analyzed, and 2020 US Census data were used for population estimates. Data were analyzed July 1, 2023, to February 1, 2024.

**Exposures:**

Adult NHANES participants enrolled between January 2017 and March 2020.

**Main Outcomes and Measures:**

Baseline weighted characteristics of HDS users and users of 6 potentially hepatotoxic botanical products were compared with non–HDS users. Multivariable analysis was undertaken to identify factors associated with HDS use or at-risk botanical use.

**Results:**

Among 9685 adults enrolled in this NHANES cohort, the mean (SE) age was 47.5 (0.5) years, and 51.8% (95% CI, 50.2%-53.4%) were female. The overall prevalence of HDS product use was 57.6% (95% CI, 55.9%-59.4%), while the prevalence of using the 6 botanicals of interest was 4.7% (95% CI, 3.9%-5.7%). Turmeric-containing botanicals were most commonly used (n = 236), followed by products containing green tea (n = 92), ashwagandha (n = 28), *Garcinia cambogia* (n = 20), red yeast rice (n = 20), and black cohosh (n = 19). Consumers of these 6 botanicals were significantly older (adjusted odds ratio [AOR], 2.36 [95% CI, 1.06-5.25]; *P* = .04 for 40-59 years of age and AOR, 3.96 [95% CI, 1.93-8.11]; *P* = .001 for ≥60 years of age), had a higher educational level (AOR, 4.78 [95% CI, 2.62-8.75]; *P* < .001), and were more likely to have arthritis (AOR, 2.27 [95% CI, 1.62-3.29]; *P* < .001) compared with non–HDS users. An estimated 15 584 599 (95% CI, 13 047 571-18 648 801) US adults used at least 1 of the 6 botanical products within the past 30 days, which was similar to the estimated number of patients prescribed potentially hepatotoxic drugs, including simvastatin (14 036 024 [95% CI, 11 202 460-17 594 452]) and nonsteroidal anti-inflammatory drugs (14 793 837 [95% CI, 13 014 623-16 671 897]). The most common reason for consuming turmeric and green tea was to improve or maintain health.

**Conclusions and Relevance:**

In this survey study, an estimated 15.6 million US adults consumed at least 1 botanical product with liver liability within the past 30 days, comparable with the number of people who consumed nonsteroidal anti-inflammatory drugs and a commonly prescribed hypolipidemic drug. Given a lack of regulatory oversight on the manufacturing and testing of botanical products, clinicians should be aware of possible adverse events from consumption of these largely unregulated products.

## Key Points

**Question:**

What percentage of US adults consume at least 1 of 6 potentially hepatotoxic botanical products?

**Findings:**

In this survey study analyzing nationally representative data from 9685 adults, 4.7% of US adults reported exposure to 6 potentially hepatotoxic botanicals: turmeric was most frequently reported, followed in order by green tea, ashwagandha, *Garcinia cambogia*, red yeast rice, and black cohosh products. Botanical product users were significantly older, more educated, and more likely to have arthritis compared with nonusers.

**Meaning:**

The results of this study suggest that clinicians should be aware of possible adverse events from consumption of these largely unregulated products.

## Teaser

This survey study assesses the prevalence of use and clinical characteristics of consumers of the 6 most frequently reported hepatoxic botanicals, including turmeric or curcumin, green tea extract, *Garcinia cambogia*, black cohosh, red yeast rice, and ashwagandha, among US adults.

## Introduction

Herbal and dietary supplements (HDSs) include a multitude of products consumed by millions of people every day to improve their general health and to treat minor ailments. Over 80 000 HDS products can be purchased without a prescription at various unregulated retail outlets or via the internet.^1^ The largest group of HDS products used include multivitamins, minerals, vitamin D, omega-3 fatty acid, and calcium with well-defined ingredients on the label. However, an estimated 5% to 12% of HDS products are plant-derived, complex multi-ingredient botanicals.^2,3^ Chemical analyses of HDS products associated with confirmed liver toxic effects show frequent discrepancies between product labels and detected ingredients.^3^ The safety and efficacy of HDSs are not well established due to the lack of regulatory requirements by the US Food and Drug Administration for human pharmacokinetic or prospective clinical trials prior to marketing.^4^

The Drug Induced Liver Injury Network (DILIN), a multicenter US observational program that collects and analyzes data from patients with hepatotoxic effects attributed to various drugs and HDS products, found that the proportion of DILI cases from HDSs nearly tripled from 7% in 2004 to 2005 to 20% in 2013 to 2014.^5,6^ The most commonly implicated botanical products in the DILIN include turmeric, kratom, green tea extract, and *Garcinia cambogia,* with potentially severe and even fatal liver injury.^7,8,9,10,11,12,13^ Furthermore, the multicenter Acute Liver Failure Study Group has also demonstrated that an increasing proportion of DILI-related acute liver failure cases were caused by HDSs, increasing from 12.4% in 1998 to 2007, to 21.1% in 2007 to 2015.^14^

The National Health and Nutrition Examination Survey (NHANES) is a periodic, population-based study of the general US population that includes comprehensive data regarding HDS use.^15^ In the current study, the proportion of NHANES patients who reported exposure to 6 potentially hepatotoxic botanicals—turmeric or curcumin, green tea, *Garcinia cambogia*, black cohosh, red yeast rice, and ashwagandha—were identified.^7,8,9,10,11,12,13^ The clinical features and baseline demographics of these individuals along with their self-reported reasons for taking these products are reviewed herein and compared with non–HDS users. To determine population level estimates of exposure to these products, US census data were used.

## Methods

### Study Population

This survey study used data from NHANES, a cross-sectional, nationally representative survey designed to monitor the health and nutrition of the civilian noninstitutionalized resident US population that has been conducted in 2-year cycles since 1999.^16^ NHANES was approved by the research ethics review board of the US Centers for Disease Control and Prevention National Center for Health Statistics, with written informed consent obtained from all adult participants. NHANES collects data from interviews, standardized physical examinations, and analyses of obtained blood and other biological specimens. Due to the COVID-19 pandemic, data collection for the NHANES 2019-2020 cycle was interrupted. Therefore, for the present analysis, data collected from January 2019 to March 2020 among adults older than 18 years of age were combined with data from the NHANES 2017 to 2018 cycle to form a nationally representative sample of NHANES 2017 to March 2020 prepandemic data.^17^ The crude response rates during 2017 to March 2020 were 51.4% for children and adolescents aged 2 to 19 years of age and 43.9% for adults aged 20 years or older.^17^ All data used in this analysis were extracted from publicly available datasets.^18^ This study followed the American Association for Public Opinion Research (AAPOR) reporting guideline for survey studies.

### NHANES Data Collection

Information on participant age, sex, race and ethnicity, marital status, educational level, family income to poverty level index, and medical history were collected through questionnaires. Race and ethnicity data were collected because of a potential difference in the prevalence of HDS use. Race and ethnicity were based on self-report and were categorized as Mexican and non-Mexican Hispanic, non-Hispanic Asian (persons having origins in any of the original peoples of East Asia, Southeast Asia, or Indian subcontinent), non-Hispanic Black or African American, non-Hispanic White, and other (eg, American Indian, Alaska Native, Native Hawaiian, Pacific Islander, >1 race, or any other race). HDS and prescription drug use data were collected through personal interviews for the 30-day period prior to the survey date. The use of HDSs reported in NHANES 2017 to 2018 and 2019 to 2020 is detailed in the NHANES Dietary Supplement Database 1999-2020.^19^ An HDS ingredient was classified as a botanical if it is part of plant, tree, shrub, or herb. We targeted our analysis to investigate national levels of exposure to the 6 most frequently implicated causes of HDS-DILI cases in the DILIN.^7,8,9,10,11,12,13^ The 6 potentially hepatotoxic botanicals of interest in our study, including turmeric or curcumin, green tea, *Garcinia cambogia*, black cohosh, red yeast rice, and ashwagandha, were identified by their ingredient and supplement identification numbers (eTable 1 in Supplement 1). However, the daily dose of an HDS product consumed by an individual patient was not recorded or available for analysis. Furthermore, confirmation of the ingredients listed on HDS product labels via analytical chemistry methods was also not available.

Approximately 95% of adults 18 years or older provided blood samples at the mobile examination centers. The blood samples were tested at central laboratories using standard protocols to determine routine laboratory parameters (eg, complete blood count, comprehensive panel) as well as fasting glycated hemoglobin, cholesterol, and triglyceride levels.

Echosens North America Vibration-Controlled Transient Elastography was performed at the mobile examination centers by NHANES technicians. Controlled attenuation parameter was used to quantify the presence and severity of hepatic steatosis. Similarly, the liver stiffness measurement score in kilopascals was used to estimate the severity of hepatic fibrosis.^20^

Self-reported chronic medical conditions that were specifically captured were current or prior history of hypertension, diabetes, coronary heart disease, stroke, arthritis, chronic obstructive pulmonary disease, thyroid disorder, cancer, and liver condition. Liver conditions included viral, autoimmune, genetic liver disease, drug- or medication-induced liver disease, alcoholic liver disease, metabolic dysfunction–associated steatotic liver disease (formerly nonalcoholic fatty liver disease), liver cyst, liver abscess, and cirrhosis. Smokers were defined as individuals who smoked 100 or more cigarettes. Regular alcohol consumption was defined as mean alcohol consumption in the past 12 months of 1 or more alcoholic beverages per day for women and 2 or more alcoholic beverages per day for men.

### Population Size Estimates

Data from the 2020 US Census were used to estimate the size with associated 95% CIs of the resident population 18 years of age or older.^21^ The prevalence of use of the 6 at risk botanicals was compared with the prevalence of widely prescribed potentially hepatotoxic medications with a LiverTox likelihood score of A or B, which included nonsteroidal anti-inflammatory drugs (NSAIDs), sertraline (antidepressant drug), and simvastatin (hypolipidemic drug).^22,23^ The NSAID prescriptions included ibuprofen, naproxen, meloxicam, celecoxib, indomethacin, ketorolac, piroxicam, and sulindac. The LiverTox likelihood score is a 5-point scale (A to E) that estimates whether a medication is a cause of liver injury: A indicates well-known cause, with more than 50 published cases; B, highly likely cause, with 12 to 49 published cases; C, probable cause, with 4 to 11 published cases; D, possible cause, with 1 to 3 published cases; E, unlikely cause; E^b^, suspected but unproven cause; and X, unknown.^23^

### Statistical Analysis

The complex survey design factors in the NHANES, including sample weights, clustering, and stratification, were accounted for as specified in the NHANES statistical analysis guideline.^17^ Baseline weighted characteristics were compared and summarized as estimated percentages or means, with a margin of error, following the AAPOR reporting guidance for survey studies.^24^ Categorical variables were compared using Fisher exact tests or χ^2^ tests if more than 2 categories existed, and continuous variables were compared using the *t* test. Multivariable analysis of factors that had a value of *P* < .10 with univariate analysis was performed to evaluate for factors associated with any HDS use as well as botanical products of interest exposure, adjusted for age group, sex, race and ethnicity, marital status, alcohol use, smoking, poverty index, and educational level. Each chronic medical condition was analyzed after adjusting for age, sex, race and ethnicity, marital status, alcohol use, smoking, income, and educational level. Odds ratios (ORs) and 95% CIs are reported. Median numbers of HDS products and prescription drugs were compared using Mann-Whitney tests. All statistical analyses were conducted from July 1, 2023, to February 1, 2024, using STATA/SE version 16.1 (StataCorp LLC). A 2-sided *P* < .05 was considered statistically significant.

## Results

### Overall HDS Use in the NHANES 2017-2020 Cohort

Among 9685 adults enrolled in this NHANES cohort, the mean (SE) age was 47.5 (0.5) years, 4971 (51.8% [95% CI, 50.2-53.4]) were female, 4714 (48.2% [95% CI, 46.6-49.8]) were male, 2121 (16.3% [95% CI, 13.5%-19.6%]) were Mexican or non-Mexican Hispanic, 1169 (5.9% [95% CI, 4.3%-8.2%]) were non-Hispanic Asian, 2552 (11.5% [95% CI, 8.8%-14.7%]) were non-Hispanic Black, 3369 (62.2% [95% CI, 57.1%-67.0%]) were non-Hispanic White, and 474 (4.1% [95% CI, 3.4%-4.8%]) were other race or ethnicity (Table 1). Overall, 5271 adults (57.6% [95% CI, 55.9%-59.4%]) reported using at least 1 HDS product within the past 30 days. HDS users were significantly older (mean [SE] age, 51.9 [0.7] vs 41.5 [0.4] years; *P* < .001) and more likely to be female (57.7% [95% CI, 55.2%-60.1%] vs 43.7% [95% CI, 42.5%-45.0]; *P* < .001), non-Hispanic White (67.6% [95% CI, 62.5%-72.4%] vs 54.8% [95% CI, 49.1%-60.3%]; *P* < .001), married (63.5% [95% CI, 59.7%-67.0%] vs 59.0% [95% CI, 57.0%-60.9%]; *P* < .001), and have a higher level of education (68.6% [95% CI, 66.0%-72.0%] vs 52.6% [95% CI, 50.9%-59.2%]; *P* < .001) compared with non–HDS users. HDS users were also less likely to smoke (39.6% [95% CI, 36.8%-42.4%] vs 44.1% [95% CI, 40.7%-47.5%]; *P* = .03) and less likely to be below the poverty line (9.5% [95% CI, 7.6%-11.8%] vs 18.3% [95% CI, 16.0%-20.7%]; *P* < .001), indicative of a higher socioeconomic status. Body mass index (calculated as weight in kilograms divided by height in meters squared; mean [SE], 29.7 [0.2] vs 29.9 [0.2]; *P* = .37) and history of alcohol use (87.2% [95% CI, 85.0%-89.0%] vs 86.3% (95% CI, 83.4%-88.8%; *P* = .63) were similar in the 2 groups.

**Table 1.** Clinical Characteristics of Adults With vs Without HDS Use in NHANES 2017-2020^a^

Characteristic	Adults, No./% (95% CI)			*P* value
	Overall (n = 9685)	With HDS use (n = 5271)	Without HDS use (n = 4414)	
Age, mean (SE), y	47.5 (0.5)	51.9 (0.7)	41.5 (0.4)	<.001
Sex				
Female	4971/51.8 (50.2–53.4)	3026/57.7 (55.2-60.1)	1945/43.7 (42.5-45.0	<.001
Male	4714/48.2 (46.6-49.8)	2245/42.3 (39.9-44.8)	2469/56.3 (55.0-57.5)	
Race and ethnicity				<.001
Mexican or Other non-Mexican Hispanic	2121/16.3 (13.5-19.6)	1013/13.2 (10.6-16.3)	1108/20.5 (16.8-24.8)	
Non-Hispanic Asian	1169/5.9 (4.3-8.2)	642/5.7 (4.0-8.0)	527/6.3 (4.6-8.7)	
Non-Hispanic Black	2552/11.5 (8.8-14.7)	1258/9.5 (7.2-12.5)	1294/14.1 (11-18)	
Non-Hispanic White	3369/62.2 (57.1-67.0)	2098/67.6 (62.5-72.4)	1271/54.8 (49.1-60.3)	
Other^b^	474/4.1 (3.4-4.8)	260/3.9 (3.1-5)	214/4.3 (3.5-5.2)	
Married	5275/61.6 (59-64.1)	3034/63.5 (59.7-67.0)	2241/59.0 (57.0-60.9)	<.001
BMI, mean (SE)	29.8 (0.2)	29.7 (0.2)	29.9 (0.2)	.37
No. of HDS products used, median (IQR)	3 (1-5)	1 (1-3)	NA	NA
≥1 Prescribed drug used	5663/58.3 (55.8-60.7)	3725/70.1 (67.7-72.4)	1938/42.2 (39.5-44.9)	<.001
No. of prescribed drugs used, median (IQR)	3 (1-5)	3 (1-6)	2 (1-4)	.27^c^
Smoked >100 cigarettes	3886/41.5 (39.1-43.8)	2070/39.6 (36.8-42.4)	1816/44.1 (40.7-47.5)	.03
Regular alcohol use^d^	4983/86.8 (85-88.3)	2664/87.2 (85.0-89.0)	2319/86.3 (83.4-88.8)	.63
Income: poverty ratio <1, No. (%)	1640/13.1 (11.4-15.1)	667/9.5 (7.6-11.8)	973/18.3 (16.0-20.7)	<.001
Some college or higher	5228/61.9 (59.8-66.4)	3269/68.6 (66.0-72.0)	1959/52.6 (50.9-59.2)	<.001
Chronic medical disorder				
Hypertension	3575/31.7 (29.4-34.1)	2300/37.1 (34.4-40)	1275/24.4 (21.9-26.9)	<.001
Diabetes	1687/13.7 (13-14.6)	1109/16.8 (15.7-18)	578/9.6 (8.4-10.9)	<.001
Coronary heart disease	421/4.2 (3.2-5.5)	294/5.1 (3.8-6.7)	127/3.03 (2.2-4.2)	.001
Stroke	486/3.8 (3.3-4.4)	312/4.5 (3.7-5.4)	174/2.8 (2.2-3.7)	.006
Arthritis	2812/27.9 (25.3-30.6)	1930/33.8 (30.4-37.5)	882/19.5 (17.7-21.4)	<.001
COPD	846/8.6 (7.6-9.8)	519/9 (7.6-10.7)	327/8.1 (6.7-9.8)	.43
Thyroid disorder	1079/12.3 (11.4-13.2)	785/16.2 (15-17.5)	294/6.8 (5.6-8.1)	<.001
Cancer	1004/11.6 (10.6-12.7)	732/15 (13.7-16.4)	272/6.8 (5.6-8.3)	<.001
Liver disorder	462/4.6 (3.9-5.3)	297/5.3 (4.5-6.4)	165/3.5 (2.8-4.4)	.002
Glycated hemoglobin, mean (SE), %	5.6 (0.0)	5.7 (0.0)	5.6 (0.0)	.007
Total cholesterol, mean (SE), mg/dL	186.9 (1.2)	188.6 (1.4)	184.4 (1.4)	.009
Triglyceride, mean (SE), mg/dL	138.9 (2.5)	135.9 (2.6)	143 (2.9)	.003
ALT, mean (SE), U/L	22.6 (0.3)	22.1 (0.3)	23.4 (0.5)	.015
AST, mean (SE), U/L	21.8 (0.2)	21.8 (0.3)	21.8 (0.2)	.98
ALP, mean (SE), U/L	75.7 (0.5)	74.5 (0.6)	77.4 (0.6)	.001
CAP score, mean (SE), dB/m	263.3 (1.4)	262.9 (1.8)	263.9 (1.5)	.54
LSM, mean (SE), kPa	5.9 (0.12)	5.8 (0.15)	5.9 (0.11)	.37

Abbreviations: ALP, alkaline phosphatase; ALT, alanine aminotransferase; AST, aspartate aminotransferase; BMI, body mass index (calculated as weight in kilograms divided by height in meters squared); CAP, controlled attenuation parameter measured in decibels per meter; COPD, chronic obstructive pulmonary disorder; HDS, herbal dietary supplement; LSM, liver stiffness measurement, in kilopascal; NA, not applicable; NHANES, National Health and Nutrition Examination Survey.

SI conversion factor: To convert HbA_1c_ percentage to proportion of total, multiply by 0.01; total cholesterol level to millimoles per liter, by 0.0259; triglyceride level to millimoles per liter, by 0.0113; ALT, AST, and ALP levels to microkatals per liter, by 0.0167.

^a^ Data are weighted characteristics and are reported as means with SEs for continuous variables and percentages with 95% CIs for categorical variables.

^b^ Other race and ethnicity included American Indian, Alaska Native, Native Hawaiian, Pacific Islander, more than 1 race, or any other race.

^c^ Mann-Whitney test.

^d^ Average alcohol consumption in the past 12 months 1 or more drinks per day for women and 2 or more drinks per day for men.

Consistent with their increased age, HDS users were significantly more likely to have hypertension, diabetes, coronary heart disease, stroke, arthritis, thyroid disorder, cancer, and liver conditions compared with non–HDS users. The median (range) number of HDS products used within 30 days was 1 (1-22). HDS users were also significantly more likely than non–HDS users to be taking a concomitant prescription medication (70.1% [95% CI, 67.7%-72.4%] vs 42.2% [95% CI, 39.5%-44.9%]; *P* < .001).

Consistent with their higher prevalence of diabetes, HDS users had significantly higher hemoglobin A_1C_ and total cholesterol levels, but they had lower triglyceride levels. Furthermore, HDS users tended to have lower serum alanine aminotransferase and alkaline phosphatase levels compared with non–HDS users (Table 1). However, there was no significant difference for controlled attenuation parameter or liver stiffness measurement scores between the 2 groups.

### Use of 6 Potentially Hepatotoxic Botanicals in NHANES 2017 to 2020

In total, 731 of 9685 US adults assessed (7.5%) used a botanical-containing HDS product within the last 30 days, and 350 participants (4.7% [95% CI, 3.9%-5.7%]) used at least 1 of the 6 botanical products of interest within the past 30 days (eFigure in Supplement 1). The most commonly used potentially hepatotoxic botanical products were turmeric or curcumin (n = 236) and green tea (n = 92), followed by ashwagandha (n = 28), *Garcinia cambogia* (n = 20), red yeast rice (n = 20), and black cohosh (n = 19). The number of unique products was 118 for turmeric, 26 for ashwagandha, 66 for green tea, 13 for *Garcinia cambogia*, 11 for black cohosh, 10 for red yeast rice, and 275 for other botanicals (eTable 2 in Supplement 1). Among 350 patients, 291 had exposure to only 1 of the 6 botanicals, 51 had exposure to 2 botanicals, and 8 had exposure to 3 or more.

Characteristics of the 6 potentially hepatotoxic botanical users (n = 350) were compared with those with no HDS use (n = 4414) (Table 2). At-risk botanical users were significantly older (mean [SE] age, 51.7 [2.0] vs 41.5 [0.4] years; *P* < .001) and more likely to be female (56.9% [95% CI, 47.7%-65.5%] vs 43.7% [95% CI, 42.5%-45.0%]; *P* = .005), non-Hispanic White (75.2% [95% CI, 65.4%-82.9%] vs 54.8% [95% CI, 49.1%-60.3%]; *P* < .001), married (66% [95% CI, 58.5%-72.7%] vs 59.0% [95% CI, 57.0%-60.9%]; *P* = .001), and have some college degree or higher (82.8% [95% CI, 76.7%-87.5%] vs 52.6% [95% CI, 50.9%-59.2%]; *P* < .001), and were less likely to be below the poverty line (5.1% [95% CI, 3.0%-8.8%] vs 18.3% [95% CI, 16.0%-20.7%]; *P* < .001). Among at-risk botanical users, the median number of HDS products used was 4 (range, 2-7) and was highest among those who consumed red yeast rice and ashwagandha (median [IQR], 7 [4-11]). Individuals who used at least 1 the 6 botanicals of interest were also more likely to be taking a prescription medication compared with non–HDS users (66.0% [95% CI, 58.9%-71.8%] vs 42.0% [95% CI, 39.5%-44.9%]; *P* < .001). Furthermore, the botanical users were more likely to have arthritis (40.0% [95% CI, 32.4%-48.1%] vs 19.5% [95% CI, 17.7%-21.4%]; *P* < .001), thyroid disorder (15.8% [95% CI, 11.0%-22.1%] vs 6.8% [95% CI, 5.6%-8.1%]; *P* = .004), and cancer (14.0% [95% CI, 9.7%-19.6%] vs 6.8% [95% CI, 5.6%-8.3%]; *P* = .006) compared with non–HDS users.

**Table 2.** Clinical Characteristics of Adults With vs Without Use of 6 Indexed Botanical Products^a^

Characteristic	Adults, No./% (95% CI)								*P* value for 6-indexed HDS vs no HDS
	Turmeric (n = 236)	Green tea (n = 92)	*Garcinia cambogia* (n = 20)	Black cohosh (n = 19)	Red yeast rice (n = 20)	Ashwagandha (n = 28)	6-Indexed HDS (n = 350)	No HDS (n = 4414)	
Age, mean (SE), y	52.2 (2.8)^b^	53.2 (2.2)^b^	44.3 (2.4)	53.2 (4.2)^b^	67.2 (2.2)^b^	51.7 (4.2)^b^	51.7 (2.0)^b^	41.5 (0.4)	<.001
Sex									
Female	132/51.2 (41.6-60.7)	54/68.3 (54.5-79.5)^b^	16/70 (36.6-90.4)	18/87.5 (42.9-98.5)^b^	12/66.8 (43.2-84.2)	14/61.9 (33.9-83.7)	202/56.9 (47.7-65.5)^b^	1945/43.7 (42.5-45.0)	.005
Male	104/48.8 (39.3-58.4)	37/31.7 (20.5-45.5)	4/29.9 (9.6-63.4)	1/12.5 (1.5-57.1)	8/33.2 (15.8-56.8)	14/38.1 (16.3-66.1)	145/43.1 (34.5-52.3)	2473/56.3 (55.0-57.5)	
Race and ethnicity									<.001
Mexican or other non-Mexican Hispanic	41/9.6 (5.6-16)	19/14.8 (7.5-27.2)	6/25.3 (7.9-57.3)	2/3 (0.6-14.4)	4/14.5 (4.6-37)	4/7 (2-21.9)	65/11.2 (6.7-18.1)	1108/20.5 (16.8-24.8)	
Non-Hispanic Asian	16/2 (1.2-3.4)	9/3.9 (1.2-12.2)	0	1/0.93 (0.1-6.4)	1/4.3 (0.4-32.4)	2/3.6 (0.7-16.2)	26/2.5 (1.6-4)	527/6.3 (4.6-8.7)	
Non-Hispanic Black	44/5.7 (3.5-9.2)	25/10.5 (5.9-17.8)	5/11.3 (18.9-45.6)	3/4.4 (1.3-13.8)	4/6.3 (2.1-17.5)	3/2.4 (0.6-8.3)	67/6 (3.8-9)	1294/14.1 (11-18)	
Non-Hispanic White	119/77.8 (68.2-85.1)^b^	36/69.8 (60-80.1)	6/40.7 (14-74)	12/90.8 (82.4-95.4)^b^	11/74.9 (48.8-90.3)	17/84.4 (67.4-93.4)^b^	168/75.2 (65.4-82.9)^b^	1271/54.8 (49.1-60.3)	
Other^c^	16/5 (2.1-11.1)	2/1.1 (0.2-5.7)	3/22.8 (6-57.5)	1/0.91 (0.1-7.3)	0	2/2.6 (1.5-4.5)	21/5.1 (2.5-10.4)	214/4.3 (3.5-5.2)	
Married	145/68.7 (60-76.3)^b^	56/65.1 (46.6-80)	10/53.4 (29.4-76)	8/53.3 (25.4-79.4)	13/67 (37.9-87.1)	16/71.9 (47.7-87.8)	208/66 (58.5-72.7)^b^	2241/59 (57-60.9)	.001
BMI, mean (SD)	28.9 (0.7)	29 (1.0)	36.2 (2.5^b^)	27.1 (2.2)	27 (0.7^b^)	26.5 (1.9)	28.9 (0.6)	29.9 (0.22)	.12
No. of HDS products used, median (IQR)	4 (2-7)	4 (2-6)	2 (2-5)	4 (2-7)	7 (5-12)	7 (4-11)	4 (2-7)	NA	NA
≥1 Prescribed drug used	165/66 (57.2-74.2)	65/76 (61.2-86.8)	11/48 (23.7-73.6)	15/69 (37.3-89.5)	15/86 (62.8-95.4)	15/73 (49-88.5)	234/66.0(58.9-71.8)	1938/42.0 (39.5-44.9)	<.001
No. of prescribed drugs used, median (IQR)	3 (1-5)	2 (1-5)	2 (1-5)	3 (2-7)	4 (1-5)	2 (1-5)	3 (1-5)	2 (1-4)	<.001^d^
Smoked >100 cigarettes	84/36.3 (27.6-46)	29/32.9 (23.3-44.2)	10/57.9 (29.9-81.6)	9/45.2 (19.9-73.3)	6/30.1 (13.5-54.4)	12/39.5 (20.4-64.5)	123/36.4 (28.6-45.2)	1819/44.1 (40.7-47.5)	.23
Alcohol use^e^	144/91.5 (83.5-95.8)	58/89.5 (71.3-96.7)	14/83.4 (36.6-97.8)	11/83.6 (33.3-98.1)	8/96.4 (72.1-99.6)	13/91.4 66.3-98.3)	210/91.4 (85.7-95)	2319/86.3 (83.4-88.8)	.06
Income: poverty ratio <1	12/4.1 (2-8.3)^b^	6/2.5(1-6.5)^b^	4/17.6 (6.8-38.6)	3/6.2 (1.1-28.7)	0^b^	3/6.8 (3.2-13.4)^b^	25/5.1 (3.0-8.8)^b^	973/18.3 (16.0-20.7)	<.001
Some college or higher	187/85.8 (78.4-91)^b^	70/85.6 (73.3-92.8)^b^	14/62.9 (31.7-86.1)	15/89.5 (67.5-97.2)^b^	16/73.5 (41.9-91.5)	24/91.4 (66.5-98.3)^b^	265/82.8 (76.7-87.5)^b^	1959/52.6 (50.9-59.2)	<.001
Chronic medical condition									
Hypertension	96/29.2 (22-37.6)	33/32.2 (20.3-47)	8/54.6 (24.9-81.4)	5/14.4 (4-40.6)	12/69.5 (40.8-88.3)^b^	9/32.4 (14.5-57.4)	132/30 (23.8-37.1)	1275/24.4 (21.9-26.9)	.06
Diabetes	42/12.1 (7.1-19.8)	20/8.3 (4.7-14.2)	5/34.2 (13.7-63.1)^b^	4/6.3 (1.7-20.9)	6/16.8 (5.8-40)	3/3.5 (1.9-6.2)^b^	67/12.7 (8.6-18.3)	578/9.6 (8.4-10.9)	.23
Coronary heart disease	8/1.9 (0.6-6.3)	3/1.6 (0.6-4.1)	0^b^	0^b^	0^b^	0^b^	10/1.6 (0.5-4.5)	127,3.03 (2.2-4.2)	.15
Stroke	9/3.7 (1.5-8.9)	3/1.7 (0.4-7.1)	0^b^	1/3.6 (0.3-29.1)	0^b^	2/3.5 (0.4-25.3)	14/3.5 (1.7-6.7)	174/2.8 (2.2-3.7)	.61
Arthritis	106/40.9 (31.7-50.7)^b^	29/33.4 (21.3-48.3)^b^	9/47.1 (23.7-71.8)^b^	9/45.8 (20.2-73.8)^b^	10/62 (38.7-80.8)^b^	8/27.6 (10-56.6)	146/40.0 (32.4-48.1)^b^	882/19.5 (17.7-21.4)	<.001
COPD	18/4.7 (2.1-10.2)	4/1.8 (0.5-6.4)^b^	1/0.67 (0.1-5.3)^b^	1/2.9 (3.7-19.8)	2/6 (0.9-32.3)	1/0.4 (0.1-3)^b^	22/4 (2-7.8)^b^	327/8.1 (6.7-9.8)	.02
Thyroid disorder	34/15.1 (9.6-23)^b^	11/20.1 (10.4-35.3)^b^	2/7.8 (1.5-32.8)	7/34.8 (13.2-65.3)^b^	6/30 (13.4-53.5)^b^	5/28 (9.8-58.1)	53/15.8 (11.0-22.1)^b^	294/6.8 (5.6-8.1)	.004
Cancer	30/13.6 (8.4-21.1)^b^	9/16.2 (7.1-32.9)^b^	2/13.6 (3.4-41.3)^b^	1/5.8 (0.7-34.6)	3/6 (1.7-18.8)	4/14.5 (3.7-42.7)^b^	44/14.0 (9.7-19.6)^b^	272/6.8 (5.6-8.3)	.006
Liver disorder	11/2.7 (1.3-5.7)	2/4.2 (0.6-23.6)	0	1/10.4 (1.3-49.8)	0	1/1.6 (0.2-11.9)	12/2.8 (1.3-6)	165/3.5 (2.8-4.4)	.51
Glycated hemoglobin, mean (SE), %	5.6 (0.1)	5.8 (0.1)	6.3 (0.5)	5.8 (0.2)	5.8 (0.2)	5.4 (0.1)^b^	5.6 (0.1)	5.6 (0.0)	.89
Total cholesterol, mean (SE), mg/dL	188.1 (5.3)	205 (4.9)^b^	179.4 (9.5)	197.8 (9.7)	217.7 (7.9)^b^	196.1 (4.8)^b^	190.7 (4.2)	184.4 (1.37)	.13
Triglyceride, mean (SE), mg/dL	122 (6.5)^b^	126.9 (10.5)	186.9 (49)	130.7 (12.6)	111.5 (12.8^b^	104.3 (17.7)^b^	126.8 (5.7)^b^	143 (2.9)	.001
ALT, mean (SE), U/L	22.7 (0.8	21.3 (0.9)	25.7 (2.2	20.5 (2)	17.3 (1.5)^b^	25.2 (3)	22.3 (0.7)	23.4 (0.5)	.20
AST, mean (SE), U/L	22 (0.6)	21.6 (0.8)	23.1 (1.6)	21 (1.8)	19 (0.6)^b^	23.3 (1.8)	21.9 (0.5)	21.8 (0.2)	.95
ALP, mean (SE), U/L	71.8 (1.3)^b^	75 (3.6)	65.5 (5.6)	69.1 (6)	66.8 (3.6)^b^	71 (4.3)	70.7 (1.2)^b^	77.4 (0.6)	<.001
CAP score, mean (SE), dB/m	255.3 (7.8)	257.6 (9.6)	283 (15.5)	235 (10.7)^b^	249.9 (11.8)	262 (12.9)	254.6 (5.9)	263.9 (1.5)	.12
LSM, mean (SE), kPa	5.2 (0.15)	4.9 (0.26)^b^	10.5 (3.8)	5.2 (0.7)	4.7 (0.2)^b^	5 (0.4)^b^	5.5 (0.25)	5.9 (0.11)	.17

Abbreviations: ALP, alkaline phosphatase; ALT, alanine aminotransferase; AST, aspartate aminotransferase; BMI, body mass index (calculated as weight in kilograms divided by height in meters squared); CAP, controlled attenuation parameter measured in decibels per meter; COPD, chronic obstructive pulmonary disease; HDS, herbal dietary supplement; LSM, liver stiffness measurement, in kilopascal; NA, not applicable.

SI conversion factor: To convert HbA_1c_ percentage to proportion of total, multiply by 0.01; total cholesterol level to millimoles per liter, by 0.0259; triglyceride level to millimoles per liter, by 0.0113; ALT, AST, and ALP levels to microkatals per liter, by 0.0167.

^a^ Data are weighted characteristics and are reported as means with SEs for continuous variables and percentages with 95% CIs for categorical variables.

^b^ *P* value significant compared with no HDS.

^c^ Other race and ethnicity included American Indian, Alaska Native, Native Hawaiian, Pacific Islander, more than 1 race, or any other race.

^d^ Mann-Whitney test.

^e^ Mean alcohol consumption in the past 12 months 1 or more drinks per day for women and 2 or more drinks per day for men.

Laboratory parameters were generally similar, but at-risk botanical users had significantly lower triglyceride and alkaline phosphatase levels (Table 2). However, there was no significant difference for glycated hemoglobin, serum aminotransferase levels or liver elastography parameters.

### Multivariable Regression Analysis

Independent factors associated with HDS use included older age (adjusted OR [AOR], 1.77 [95% CI, 1.36-2.32]; *P* < .001 for 40-59 years; AOR, 3.97 [95% CI, 2.99-5.28]; *P* < .001 for ≥60 years,), female sex (AOR, 1.76 [95% CI, 1.42-2.18]; *P* < .001), non-Hispanic White race and ethnicity (AOR, 1.25 [95% CI, 1.04-1.49]; *P* = .02), poverty ratio higher than 1 (AOR, 1.41 [95% CI, 1.11-1.77]; *P* = .006), and some college education (AOR, 2.02 [95% CI, 1.61-2.52]; *P* < .001) (Table 3). In addition, the presence of hypertension (AOR, 1.37 [95% CI, 1.11-1.70]; *P* < .001), diabetes (AOR, 1.55 [95% CI, 1.21-1.97]; *P* < .001), arthritis (AOR, 1.31 [95% CI, 1.13-1.52]; *P* = .001) and thyroid disorder (AOR, 1.60 [95% CI, 1.13-2.25]; *P* = .01) remained significantly associated with HDS use after adjusting for age, sex, race and ethnicity, marital status, smoking, income, and education level. Features associated with at-risk botanical users included older age (AOR, 2.36 [95% CI, 1.06-5.25]; *P* = .04 for 40-59 years of age; AOR, 3.96 [95% CI, 1.93-8.11]; *P* = .001 for age ≥60 years), some college education (AOR, 4.78 [95% CI, 2.62-8.75]; *P* < .001), and the presence of arthritis (AOR, 2.27 [95% CI, 1.62-3.29]; *P* < .001) after adjusting for other covariates (Table 3).

**Table 3.** Multivariable Analysis for Factors Associated With HDS Use or With 6 Indexed Botanical Products vs No HDS Use^a^

Variable	HDS vs no HDS, AOR (95% CI)	*P* value	6 Indexed HDS vs no HDS, AOR (95% CI)	*P* value
Age, y				
40-59	1.77 (1.36-2.32)	<.001	2.36 (1.06-5.25)	.04
≥60	3.97 (2.99-5.28)	<.001	3.96 (1.93-8.11)	.001
Female	1.76 (1.42-2.18)	<.001	1.61 (0.99-2.61)	.06
Non-Hispanic White	1.25 (1.04-1.49)	.02	1.38 (0.80-2.35)	.23
Married	0.93 (0.77-1.12)	.45	0.94 (0.63-1.41)	.77
Alcohol use^b^	0.99 (0.72-1.38)	.98	1.82 (0.90-3.68)	.09
Smoker^c^	1.17 (0.94-1.47)	.16	0.94 (0.51-1.72)	.83
Poverty index ratio >1	1.41 (1.11-1.77)	.006	2.15 (0.90-5.16)	.08
Some college or above	2.02 (1.61-2.52)	<.001	4.78 (2.62-8.75)	<.001
Hypertension	1.37 (1.11-1.70)	<.001	1.18 (0.73-1.91)	.50
Diabetes	1.55 (1.21-1.97)	.001	1.43 (0.81-2.52)	.20
Arthritis	1.31 (1.13-1.52)	.001	2.27 (1.62-3.29)	<.001
Coronary heart disease	1.55 (0.91-2.66)	.10	0.45 (0.09-2.34)	.33
Stroke	1.22 (0.83-1.78)	.30	0.97 (0.32-2.97)	.96
COPD	0.87 (0.59-1.29)	.48	0.51 (0.20-1.30)	.15
Thyroid disorder	1.60 (1.13-2.25)	.01	1.32 (0.84-2.06)	.21
Cancer	1.18 (0.80-1.74)	.39	1.10 (0.55-2.20)	.79
Liver condition	1.58 (0.76-3.27)	.21	0.65 (0.22-1.90)	.42

Abbreviations: AOR, adjusted odds ratio; COPD, chronic obstructive pulmonary disorder; HDS, herbal and dietary supplements.

^a^ Medical condition was adjusted for age, sex, race and ethnicity, marital status, income, educational level, and smoking for HDS vs no HDS, and adjusted for age, sex, race and ethnicity, marital status, income, and educational level for 6 indexed botanical products vs no HDS.

^b^ Mean alcohol consumption in the past 12 months 1 or more drinks per day for women and 2 or more drinks per day for men.

^c^ Smoked more than 100 cigarettes.

### Stated Reasons for Using the 6 Potentially Hepatotoxic Botanicals

**Figure 1.**
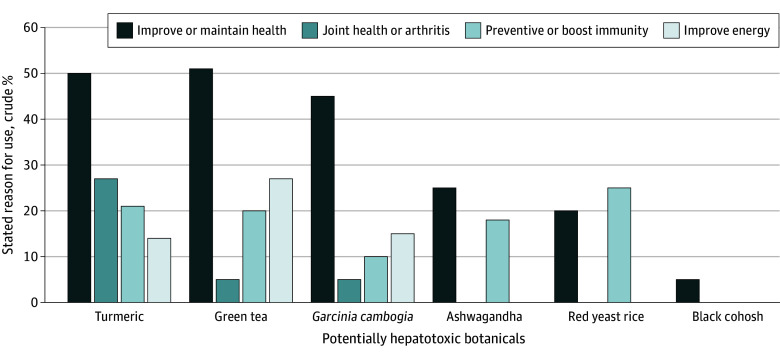
Self-Reported Reasons for Consuming 6 Potentially Hepatotoxic Botanical Products

The vast majority of at-risk botanical users were doing so of their own accord, and use of these products was not recommended by their health care providers (87.6% [95% CI, 82.8%-91.9%] for turmeric; 80.4% [95% CI, 63.8%-92.8%] for green tea; 100% for *Garcinia cambogia*; 63.2% [ 95% CI, 23.5%-82.5%] for black cohosh; 40% [95% CI, 39.9%-92.2%] for red yeast rice; and 96.4% [95% CI, 75.8%-99.7%] for ashwagandha). The most common reasons for using the botanical were to improve or maintain health or to prevent health problems or boost immunity (Figure 1). In addition, 64 turmeric users (26.8%) consumed those products for joint health or arthritis, and 25 green tea users (27.2%) were trying to improve their energy level. In total, 14 *Garcinia cambogia* users (70.0%) were trying to lose weight and had the highest median body mass index and proportion with diabetes (34.2% [95% CI, 13.7%-63.1%]) (Table 2). Similarly, 84.2% of black cohosh users were taking these products to treat hot flashes, and the vast majority of these patients were women (87.5% [95% CI, 42.9%-98.5%]). The main stated reason to consume red yeast rice was for heart health (90.0%), and these individuals tended to be older and had the second highest incidence of diabetes.

### US Population Estimates of Exposure to Botanicals of Interest

Extrapolating from the NHANES data, we observed approximately 4.7% (95% CI, 4.0%-5.7%), or an estimated 15 584 599 (95% CI, 13 047 571-18 648 801), of US adults used at least 1 of the 6 potentially hepatotoxic botanical products within the past 30 days. The most common products used were turmeric or curcumin, estimated at 11 400 151 (95% CI, 906 813-14 332 559) adults, and green tea, estimated at 3 327 790 (95% CI, 2 306 389-4 777 520) adults. An estimated 1 252 040 (95% CI, 757 813-2 075 750) adults used ashwagandha, 1 219 091 (95% CI, 724 865-32 075 750) adults used black cohosh, 889 607 (95% CI, 494 226-1 581 524) adults used *Garcinia cambogia*, and 626 020 (95% CI, 362 433-1 120 246) adults used red yeast rice.

**Figure 2.**
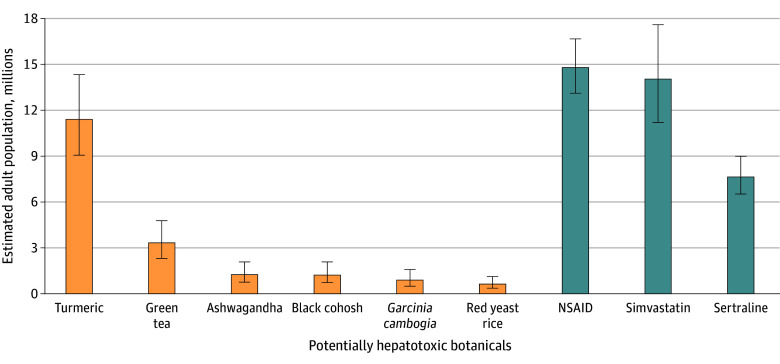
United States Population Estimates for Use of 6 Potentially Hepatotoxic Botanical Products Compared With 3 Commonly Prescribed Medications Data are based on 2020 US Census estimated total resident population 18 years of age or older of 329 484 123 July 1, 2020.^21^ Whiskers represent 95% CI. NSAIDs indicates nonsteroidal anti-inflammatory drugs.

The prevalence of using the 6 potentially hepatotoxic botanical products was compared with the prevalence of the use of known hepatotoxic prescription medications (LiverTox class A or B) that are used for similar indications. Approximately 14 793 837 (95% CI, 13 014 623-16 671 897) US adults used prescription nonsteroidal anti-inflammatory drugs, which are typically used for indications similar to those for turmeric (ie, pain or arthritis). Simvastatin, a hypolipidemic drug used to treat and prevent cardiovascular disease similar to the use for red yeast rice, was consumed by 14 036 024 (95% CI, 11 202 460-17 594 452) individuals. The prevalence of sertraline use was 7 676 980 (95% CI, 6 523 786-8 994 917) individuals. The comparison of the botanical products of interest with commonly prescribed prescription medications is shown in Figure 2 (based on the 2020 US Census estimated total resident population 18 years of age or older of 329 484 123, July 1, 2020^21^).

## Discussion

This survey study assessed the prevalence and clinical characteristics of consumers of the 6 most frequently reported hepatoxic botanicals, including turmeric or curcumin, green tea extract, *Garcinia cambogia*, black cohosh, red yeast rice, and ashwagandha, in a representative sample of US adults. We estimated that at least 15.6 million US adults used at least 1 of 6 potentially hepatotoxic botanical products within the past 30 days, which was similar to estimated number of US adults prescribed an NSAID or simvastatin.

The Dietary Supplement Health and Education Act (DSHEA) of 1994 defined an HDS as a product that contains a “dietary ingredient,” such as vitamins, minerals, herbs or botanicals, amino acids, and dietary substances that are intended to supplement the diet.^25^ HDS use has dramatically increased over time in the United States. from 32.9% in the NHANES I cohort (1971-1974) to 52% in the NHANES 2011-2012 cohort and 57.6% in the NHANES 2017-2018 cohort.^2,26^ The economic impact of the HDS products industry in the US is profound, with over $150 billion in marketplace sales in 2023, and rivals that of all prescription drugs combined.^27^ The most common HDSs used are multivitamins or minerals, calcium, fish oil, botanical supplements, and vitamin C.^28,29^ In the US, a variety of adverse events related to HDS use have been described, with an estimated 23 000 annual emergency department visits and 2154 hospitalizations in 2014.^30^ The incidence of HDS-DILI is also increasing over time and accounts for over 20% of cases of liver injury recorded in the DILIN prospective registry.^7^ The HDS-DILI can be not only severe, leading to hepatocellular injury with jaundice, but also fatal, leading to death or liver transplantation.^31,32,33,34,35,36,37^ Kesar et al^35^ reported the number of liver transplants due to HDS-DILI in 2010 through 2020 increased over 70% when compared with 1994 through 2009.

The prevalence of the use of potentially hepatotoxic botanical products rather than use of benign, non-hepatotoxic HDSs, such as vitamins and minerals, has not been systematically studied nor reported. The present study found that between January 2017 and March 2020, approximately 15 million US adults consumed at least 1 potentially hepatotoxic botanical product within the past 30 days, which was comparable to the number of people taking potentially hepatotoxic prescription drugs, such as simvastatin, NSAIDs, and sertraline (Figure 2).

The clinical characteristics of the users of the 6 botanical products of interest were similar to overall HDS users, with older age, more women and non-Hispanic White individuals, higher income, and higher level of education among HDS users compared with non–HDS users.^38,39,40^ Use of HDSs has also been shown to be more prevalent among individuals with chronic medical conditions, including cardiovascular disorders, cancer, and obesity.^26,40,41,42,43,44,45,46,47^ In our study, we found diabetes, arthritis, and thyroid disorder independently associated with HDS use, but not cardiovascular disorder or cancer. Arthritis was independently associated with the use of the 6 potentially hepatotoxic botanical products and with overall HDS use (Table 3).

The reasons for botanical use varied substantially by the specific products as well as the age, gender, and demographic features of the individual product users (Table 2 and Figure 1). For example, turmeric-containing products were most commonly used for joint health or arthritis due to the widespread belief that turmeric may have antioxidant and anti-inflammatory properties as touted in ayurvedic medicine.^48^ However, multiple randomized clinical trials have failed to demonstrate any efficacy of turmeric-containing products in osteoarthritis.^48,49^ Green tea–containing products were mostly used as energy supplements. However, multiple studies have failed to demonstrate any objective evidence of weight loss and sustained improvement in mood or energy levels with products that contain high levels of catechins or polyphenols found in green tea extract.^50,51,52^ *Garcinia cambogia* was commonly used for weight loss (70%), as it has been touted that hydroxycitric acid promotes weight loss.^53^ Black cohosh was used for hot flashes, and ashwagandha was used as muscle builder.

In the United States, HDSs are regulated under the general umbrella of foods and are not intended to be taken for disease treatment or prevention.^54,55^ Assuming that HDS products are generally safe similar to foods, the FDA does not require manufacturers to verify the ingredients in a given product or lot. But recent studies by DILIN have shown substantial discrepancies between product labels and the results of mass spectroscopy of the actual products.^3^ In addition, human bioavailability and safety studies are not required prior to the marketing of an HDS product unless the formulation contains a novel chemical entity that was not known prior to 1994. The active ingredients and components in botanical products are even more challenging to standardize due to the impact of changes in soil, local environment, and batch to batch variation in plant or cultivar production. The majority of the at-risk botanical users in this study consumed these products without clinician recommendations presumably due to the touted benefits of the products being marketed. The number of HDS products marketed in the US increased from 4000 in 1993 to 55 000 in 2012, and approximately 80 000 products were available by 2022.^1,56^

### Limitations

Our study has several important limitations. First, the survey response rate for the January 2017 to March 2020 prepandemic cohort was low, at 43.9% for adults aged 20 years or older. Since NHANES is a cross-sectional study, there was no opportunity to determine associations with clinical outcomes, such as episodes of idiosyncratic hepatotoxic effects. In addition, this survey sample size was not adequate to detect hepatotoxic effects from botanicals or other adverse events since these arise in less than 1% of exposed individuals. Thus, our study was not designed to identify any causal relationship between consumption of the 6 botanicals of interest and the development of liver injury over time. Lastly, use of HDS products and medications was obtained by self-report in NHANES and not independently verified by source documents. The ingredients data used in this study may be limited in accuracy due to poor governmental regulation and confirmation of the ingredients listed on the product label, given that previous analysis has shown discrepancies between product labels and detected ingredients.^3^ However, NHANES is the largest available nationally representative database with detailed information regarding dietary supplement product usage in the United States.

## Conclusions

This survey study found that in the NHANES 2017 to March 2020 study, over 7% of US adults used a botanical-containing HDS product within the last 30 days and that the 6 products most commonly implicated in liver injury in the US are popular among US adults and used as frequently as common hypolipidemic drugs, NSAIDs and antidepressants. In light of the lack of regulatory oversight on the manufacturing and testing of botanical products, it is recommended that clinicians obtain a full medication and HDS use history when evaluating patients with unexplained symptoms or liver test abnormalities. Considering widespread and growing popularity of botanical products, we urge government authorities to consider increasing the regulatory oversight on how botanicals are produced, marketed, tested, and monitored in the general population.

## Notes

Supplement 1. **eTable 1.** Ingredient Identification Number and Supplement Identification Number Identified the 6 Potential Hepatotoxic Botanical Products **eTable 2.** Botanical Products by Name (Alphabetical) **eFigure.** Herbal and Dietary Supplement Use Among U.S. Adults Enrolled in NHANES 2017-2020

Supplement 2.
Data Sharing Statement
