# Supplementary material for: Estimated Exposure to 6 Potentially Hepatotoxic Botanicals in US Adults
Source: JAMA Netw Open. 2024 Aug 5;7(8):e2425822. doi: 10.1001/jamanetworkopen.2024.25822 (PMC11301549; doi:10.1001/jamanetworkopen.2024.25822)
Supplement: Supplement 1. — eTable 1. Ingredient Identification Number and Supplement Identification Number Identified the 6 Potential Hepatotoxic Botanical Products eTable 2. Botanical Products by Name (Alphabetical) eFigure. Herbal and Dietary Supplement Use Among U.S. Adults Enrolled in NHANES 2017-2020 [file jamanetwopen-e2425822-s001.pdf]

Supplementary Online Content

Likhitsup A, Chen VL, Fontana RJ, et al. Estimated exposure to 6 potentially hepatotoxic botanicals in US adults. *JAMA Netw Open*. 2024;7(8):e2425822. doi:10.1001/jamanetworkopen.2024.25822

eTable 1. Ingredient Identification Number and Supplement Identification Number Identified the 6 Potential Hepatotoxic Botanical Products

eTable 2. Botanical Products by Name (Alphabetical)

eFigure. Herbal and Dietary Supplement Use Among U.S. Adults Enrolled in NHANES 2017-2020

This supplementary material has been provided by the authors to give readers additional information about their work.

© 2024 Likhitsup A et al. *JAMA Network Open*.

eTable 1. Ingredient Identification Number and Supplement Identification Number Identified the 6 Potential Hepatotoxic Botanical Products

	Ingredient identification number	supplement identification number
Turmeric/Curcumin	10000454, 10000469, 10000481, 10000629, 1001307, 10002733, 10002959, 10003127, 10003219, 10004382, 10004581, 10005542, 10006720, 10007731, 10008191, 10008201, 10008216, 10008966, 10009076, 10009077, 10009475, 100009519, 10009996, 10010066, 10010815, 10011115, 10011136, 10011464, 10011624, 10011723, 10011909	17816, 17982, 17984, 18001, 18020, 18049, 18169, 18259, 18325, 18348, 18395, 18400, 18414, 18442, 18443, 18449, 18486, 18516, 18519, 18533, 18593, 18594, 18674, 18686, 18716, 18724, 18788, 18807, 18842, 18862, 18867, 18885, 18907, 18912, 18914, 18940, 18978, 18982, 19025, 19041, 19086, 19126, 19195, 19203, 19206, 19571, 19597, 19602, 19671, 19674, 19679, 19685, 19687, 19721, 19749, 19752, 19753, 19777, 19784, 19795, 19818, 19828, 19834, 19866, 19871, 19875, 19876, 19877, 19978, 19998, 20013, 20015, 20117, 20140, 200143, 20174, 20188, 20225, 20226, 20319, 20321, 20335, 20405, 20446, 20447, 20462, 20478, 20530, 20533, 20588, 20591, 20604, 20674, 20680, 20683, 20713, 20720, 20722, 20731, 20744, 20745, 20858, 20901
Green Tea Extract	10000086, 10000449, 10000516, 10000784, 10001082, 10001288, 10001148, 10002453, 10002775, 10002851, 10002916, 10002955, 10003209, 10003275, 10003447, 10003748, 10003767, 10003877, 10004001, 10006474, 10006582, 10006586, 10006742, 10006705, 10007604, 10007605, 10008119, 10009948, 10010818	15365, 15383, 16480, 17769, 17783, 17789, 17875, 18020, 18043, 18073, 18163, 18359, 18466, 18518, 18545, 18832, 18856, 18868, 18877, 18882, 18919, 18955, 19036, 19179, 19196, 19199, 19366, 19422, 19460, 19462, 19504, 19536, 19674, 19713, 19777, 19824, 19834, 19870, 19873, 19884, 19966, 19972, 19981, 19983, 20151, 20173, 20225, 20319, 20325, 20335, 20364, 20408, 20540, 20601, 20690, 20702, 20714, 20722, 20725, 20755, 20901
Garcinia cambogia	10000654, 10000697, 10001092, 10002458, 10006459, 10006527, 10010332, 10010834, 10011071, 10011493	17832, 17955, 19180, 19667, 19674, 19733, 20009, 20400, 20634
Black Cohosh	10000043, 10000044, 10000445, 10000764, 10000897, 10001062, 10002264, 10002267, 10002535, 10004682, 10006540, 10007474, 10007475, 10008239, 10010821, 10011444	17897, 18104, 18180, 18506, 19418, 20266, 20305, 20364
Red Yeast Rice	10004356	6750, 8490, 19712, 20291
Ashwagandha	10001370, 10002263, 10003249, 1004021, 10004587, 10006560, 10006683, 10007285, 10008775, 10010377, 10010805, 10010836, 10011073, 10011732	17819, 18012, 18025, 18079, 18458, 18600, 18718, 18779, 19070, 19072, 19176, 19354, 19537, 19997, 20033, 20089, 20175, 20181, 20225, 20293, 20340, 20432, 20435, 20467, 20562, 20581, 20651, 20841

© 2024 Likhitsup A et al. *JAMA Network Open*.

eTable 2. Botanical Products by Name (Alphabetical)

TURMERIC/CURCUMIN
ANDREW LESSMAN PROCAPS LIVER ANTI-OXIDANT EXTRACTS MILK THISTLE (SILYMARIN) TURMERIC (CURCUMIN) ARTICHOKE
ANIMAL PAK THE TRUE ORIGINAL THE ULTIMATE TRAINING PACK
ASQUARED NUTRITION TURMERIC CURCUMIN WITH BIOPERINE 95% CURCUMINOIDS 1300 MG 100% NATURAL
BEYOND RAW RAW ELEMENTS A.M./P.M. VITAPAK
BOTANIC CHOICE ESSENTIALS TURMERIC 500 MG.
CUR-Q10 ULTRA HIGH ANTIOXIDANT CURCUMIN COQ10 COMPLEX
CVS HEALTH TURMERIC COMPLEX 500 MG WITH BLACK PEPPER
CVS HEALTH TURMERIC CURCUMIN 500 MG
DEFAULT CURCUMIN
DEFAULT TURMERIC
DOCTOR'S BEST HIGH ABSORPTION CURCUMIN FROM TURMERIC ROOT WITH CURCUMIN C3 COMPLEX AND BIOPERINE 500 MG
FINEST NUTRITION TURMERIC 450 MG PER CAPSULE ONE PER DAY
FINEST NUTRITION TURMERIC 500 MG PER CAPSULE
FINEVINE TURMERIC CURCUMIN WITH GINGER AND BIOPERINE BLACK PEPPER
GAIA HERBS CURCUMIN SYNERGY TURMERIC SUPREME EXTRA STRENGTH
GAIA HERBS TURMERIC SUPREME EXTRA STRENGTH
GARDEN OF LIFE DR. FORMULATED ENZYMES ORGANIC DIGEST+ 29 ORGANIC FRUITS & VEGGIES+ 120,000 UNITS OF PAPAIN CHEWA..
GARDEN OF LIFE MYKIND ORGANICS CERTIFIED ORGANIC WHOLE FOOD HAIR, SKIN & NAILS ORGANIC PLANT COLLAGEN BUILDER WH..
GNC AMP ADVANCED MUSCLE PERFORMANCE MEN'S STRENGTH VITAPAK PROGRAM INCLUDES CLINICALLY STUDIED MULTIVITAMIN BLEND
GNC HERBAL PLUS TURMERIC CURCUMIN 500 MG VEGETARIAN STANDARDIZED EXTRACT
GNC MEGA MEN 50 PLUS CLINICALLY STUDIED MULTIVITAMIN TIMED-RELEASE NEW! SMALLER PILLS
GNC MEGA MEN 50 PLUS VITAPAK PROGRAM WITH CLINICALLY STUDIED MULTIVITAMIN
GNC MEGA MEN CLINICALLY STUDIED MULTIVITAMIN TIMED-RELEASE
GNC MEGA MEN ENERGY & METABOLISM CLINICALLY STUDIED MULTIVITAMIN TIMED-RELEASE NEW! SMALLER PILLS
GNC MEGA MEN ENERGY & METABOLISM VITAPAK PROGRAM WITH CLINICALLY STUDIED MULTIVITAMIN BLEND
GNC MEGA MEN PERFORMANCE & VITALITY VITAPAK PROGRAM WITH CLINICALLY STUDIED MULTIVITAMIN BLEND
GNC MEGA MEN SPORT CLINICALLY STUDIED MULTIVITAMIN TIMED-RELEASE NEW! SMALLER PILLS
GNC TRIFLEX FAST-ACTING CLINICAL-STRENGTH DOSES OF GLUCOSAMINE/CHONDROITIN & BOSWELLIA PLUS TURMERIC
GNC WOMEN'S ULTRA MEGA 50 PLUS CLINICALLY STUDIED MULTIVITAMIN TIMED-RELEASE NEW! SMALLER PILLS
GNC WOMEN'S ULTRA MEGA ACTIVE CLINICALLY STUDIED MULTIVITAMIN TIMED-RELEASE NEW! SMALLER PILLS
GNC WOMEN'S ULTRA MEGA CLINICALLY STUDIED MULTIVITAMIN TIMED-RELEASE
GNC WOMEN'S ULTRA MEGA ENERGY & METABOLISM CLINICALLY STUDIED MULTIVITAMIN TIMED-RELEASE NEW! SMALLER PILLS
GNC WOMEN'S ULTRA MEGA WITHOUT IRON & IODINE CLINICALLY STUDIED MULTIVITAMIN TIMED-RELEASE
HEALTH PLUS KIDNEY CLEANSE 550 MG EACH KEY INGREDIENTS BUCHU LEAF EXTRACT CRANBERRY EXTRACT 50:1 TURMERIC ROOT C..

HIMALAYA TURMERIC95 WITH CURCUMIN

IMMUNOTEC OMEGA GEN V OMEGA-3 COQ10 VIT. E CURCUMA PIPERINE
INNOVIX LABS HIGH ABSORPTION CURCUMIN TURMERIC EXTRACT TIME RELEASE FORMULA BIO-ACTIVE EXTRACT OF TURMERIC

INSTAFLEX ADVANCED FEATURING UC-II COLLAGEN

JARROW FORMULAS CURCUMIN 95 TURMERIC EXTRACT 500 MILLIGRAMS

JOINT FORMULA HEALTHY JOINT FORMULA

LIFE EXTENSION SUPER BIO-CURCUMIN 400 MG
LIVEWELL MAXIMUM STRENGTH CURCUWELL HIGH-POTENCY CURCUMIN AND BOSWELLIA BLEND 1300 MG FORMULA INCLUDES BIOPERINE..

LIVINGWELL NUTRACEUTICALS HEAL-N-SOOTHE SYSTEMIC ENZYME FORMULA
MEGAFOOD TURMERIC STRENGTH FOR WHOLE BODY WITH TURMERIC, BLACK PEPPER & TART CHERRY VITAMIN & HERBAL
METHYL GENETIC NUTRITION BY PROFESSIONAL HEALTH PRODUCTS GLUTAMATE SCAVENGER/ CALMING FORMULA PROVIDES A NATURAL..
MSQUARE OKINAWA TRIPLE TURMERIC+ BLEND OF 3 TURMERIC VARIETALS + WITH BLACK PEPPER 766 MG TURMERIC 100 MG CURCUMIN

NATURE MADE TURMERIC CURCUMIN

NATURE'S BOUNTY STANDARDIZED EXTRACT TURMERIC 500 MG STANDARDIZED TO CONTAIN 93% CURCUMINOIDS

NATURE'S BOUNTY TURMERIC 450 MG PLUS TURMERIC EXTRACT 50 MG STANDARDIZED FOR 95% CURCUMINOIDS

NATURE'S BOUNTY TURMERIC 538 MG STANDARDIZED EXTRACT

NATURE'S NUTRITION TURMERIC & GINGER WITH BIOPERINE 1950 MG W/ BIOPERINE

NATURE'S NUTRITION TURMERIC CURCUMIN WITH BIOPERINE 1950 MG W/ BIOPERINE
NATURE'S TRUTH VITAMINS ADVANCED TURMERIC CURCUMIN COMPLEX 1500 MG PER SERVING PLUS BLACK PEPPER EXTRACT OLIVE L..

NATURE'S WAY STANDARDIZED TURMERIC 95% CURCUMINOIDS

NATURECITY TRUECURCUMIN HIGHLY ABSORBABLE BCM-95
NATUREWISE CURCUMIN MADE WITH ORGANIC CURCUMIN AND 95% CURCUMINOIDS 2250 MG PER SERVING WITH ORGANIC GINGER & BI..
NEW CHAPTER 40+ EVERY WOMAN'S ONE DAILY MULTI WHOLE-FOOD FERMENTED MUTIVITAMIN MADE WITH ORGANIC VEGETABLES AND ..
NEW CHAPTER EVERY MAN MULTIVITAMIN WHOLE-FOOD CULTURED MULTIVITAMIN MADE WITH ORGANIC VEGETABLES AND HERBS
NEW CHAPTER EVERY MAN'S ONE DAILY MULTI COMPLETE WHOLE-FOOD MULTIVITAMIN MADE WITH ORGANIC VEGETABLES & HERBS
NEW CHAPTER EVERY WOMAN'S ONE DAILY MULTI WHOLE-FOOD CULTURED MULTIVITAMIN WITH NUTRIENTS MADE WITH ORGANIC VEGE..
NEWVITALITY RUBY REDS POMEGRANATE, ACAI & MAQUI A DELICIOUS FRUIT AND VEGETABLE WITH POTENT VITAMINS, MINERALS, ..

NOW CURCUMIN SOFTGELS FROM TURMERIC ROOT EXTRACT STANDARDIZED EXTRACT 95% CURCUMINOIDS

NOW LIVER REFRESH

NUTRILITE DOUBLE X 22 VITAMINS AND MINERALS AND 22 PLANT CONCENTRATES 1 PACKET, 2X A DAY
NUTRILITE PERFECT PACK WITH 22 VITAMINS AND MINERALS, AND MORE THAN 22 PLANT CONCENTRATES INCLUDES DOUBLE X 2 PA..

OPTIM NUTRITION TURMERIC PLUS WITH BIO-ABSORB CURCUWIN

OREGON'S WILD HARVEST TURMERIC USDA ORGANIC

ORGANIC INDIA TURMERIC FORMULA

© 2024 Likhitsup A et al. *JAMA Network Open*.

OSTEO BI-FLEX JOINT HEALTH TRIPLE STRENGTH +TURMERIC GLUCOSAMINE TURMERIC WITH JOINT SHIELD 2 PER DAY
PHYSICIAN'S CHOICE TURMERIC WITH BOSWELLIA, GINGER & BIOPERINE STANDARDIZED 95% CURCUMINOIDS
PLANTORIGIN TURMERIC CURCUMIN WITH BIOPERINE 1500 MG HIGHEST POTENCY OF 95% CURCUMINOIDS
PLNT TURMERIC CURCUMA LONGA
PURE BY NATURE TURMERIC CURCUMIN WITH BLACK PEPPER EXTRACT 1,200 MG TURMERIC PER SERVING
PURE ENCAPSULATIONS ULTRANUTRIENT WITH METAFOLIN L-5-MTHF; MULTIVITAMIN/MINERAL FORMULA WITH ENHANCED SUPPORT
PURITAN'S PRIDE TURMERIC 400 MG NATURAL WHOLE HERB
PURITAN'S PRIDE TURMERIC 800 MG NATURALLY CONTAINS CURCUMIN NATURAL WHOLE HERB
PURITAN'S PRIDE TURMERIC CURCUMIN 500 MG WITH 50 MG OF TURMERIC EXTRACT STANDARDIZED TO CONTAIN 95% CURCUMINOIDE..
QUNOL EXTRA STRENGTH TURMERIC CURCUMIN COMPLEX ULTRA HIGH ABSORPTION 1000 MG
RADIANCE TURMERIC 400 MG
RAINBOW LIGHT ADVANCED ENZYME SYSTEM RAPID RELEASE FORMULA PLANT-SOURCE ENZYMES & SOOTHING HERBS
REAL HEALTH PROSTATE COMPLETE MULTI-ACTION FORMULA ONE-PER-DAY NATURAL BIOACTIVE SOURCE OF BETA SITOSTEROL
REXALL NATURALIST TURMERIC CURCUMIN 500 MG WITH 50 MG OF TURMERIC EXTRACT STANDARDIZED TO CONTAIN 95% CURCUMINOIDS
ROOT2 TURMERIC EXTRACT CURCUMIN C3 COMPLEX 1,160 MG PER SERVING WITH BIOPERINE
SCHWARTZ BIORESEARCH PREMIUM ULTRA PURE TURMERIC CURCUMIN WITH BIOPERINE 1500 MG 95% STANDARDIZED CURCUMINOIDS
SCIENCE NATURAL SUPPLEMENTS TURMERIC WITH BIOPERINE 1300 MG TURMERIC EXTRACT PER SERVING MAX ABSORPTION
SMARTER NUTRITION CURCUMIN 95% TETRA-HYDRO CURCUMINOIDS
SOLGAR FULL SPECTRUM CURCUMIN
SOURCE NATURALS LIFE FORCE MULTIPLE
SPRING VALLEY STANDARDIZED EXTRACT TURMERIC CURCUMIN 500 MG PER CAPSULE WITH 50 MG GINGER POWDER
SPRING VALLEY STANDARDIZED TURMERIC CURCUMIN COMPLEX
SPRING VALLEY SUPERIOR BIOAVAILABILITY STANDARDIZED TURMERIC CURCUMIN COMPLEX WITH CURCUWIN 550 MG PER SERVING
SPRING VALLEY WHOLE HERB TURMERIC CURCUMIN 500 MG PER CAPSULE
STARLIGHT LIFEGUARD JOINT FORMULA
SUNDOWN NATURALIST TURMERIC CURCUMIN 500 MG WITH 50 MG OF TURMERIC EXTRACT STANDARDIZED TO CONTAIN 95% CURCUMINO..
SWANSON PREMIUM BRAND TURMERIC 720 MG
SYNERGY THERACURMIN TURMERIC EXTRACT 300 MG
THE VITAMIN SHOPPE TURMERIC EXTRACT 300 MG 95% CURCUMINOIDS
THE VITAMIN SHOPPE TURMERIC WITH CURCUMIN 500 MG 95% CURCUMINOIDS
TNVITAMINS TURMERIC CURCUMIN 500 MG WITH BLACK PEPPER EXTRACT
TRADER JOE'S TURMERIC STANDARDIZED EXTRACT
TRUE HEALTH DR. CUTLER'S LIVER & KIDNEY CLEANSE
TURMERIC 500 MG UP&UP
UPWELLNESS GOLDEN REVIVE+ ADVANCED FORMULA

© 2024 Likhitsup A et al. *JAMA Network Open*.

VIBRANT HEALTH MAXIMUM VIBRANCE CONTAINS ALL KNOWN NUTRIENTS PLANT-BASED MULTI CLINICALLY FORMULATED VERSION 3..
VIBRANT HEALTH MAXIMUM VIBRANCE CONTAINS ALL KNOWN NUTRIENTS PLANT-BASED MULTI- ADVANCED DAILY FUTUREFOOD CLINIC..
VIMERSON HEALTH TURMERIC & GINGER BIOPERINE
VIMERSON HEALTH TURMERIC CURCUMIN BIOPERINE
VIMERSON HEALTH TURMERIC CURCUMIN MADE WITH ORGANIC TURMERIC POWDER
VITABREEZE GLUCOSAMINE CHONDROITIN MSM & TURMERIC TRIPLE STRENGTH FORMULA
VITALITY WORKS 100% VEGETARIAN TURMERIC PLUS PM ACHE RELIEF
VITAMIN WORLD TURMERIC CURCUMIN CURCUMA LONGA 500 MG WITH 50 MG OF TURMERIC EXTRACT STANDARDIZED TO CONTAIN 95% ..
WALGREENS TURMERIC 500 MG
WHOLE FOODS MARKET FOOD-CULTURED MEN'S +40 MULTI A ONE TABLET DAILY FOOD-CULTURED FORMULA WITH TARGETED HERBS & ..
WINDMILL NATURAL VITAMINS BLUEPRINT STANDARDIZED HERBALS TURMERIC 95% CURCUMINOIDS
YOUTHEORY 1,000 MG TURMERIC EXTRA STRENGTH FORMULA 1,000 MG PER DAILY SERVING
YOUTHEORY TURMERIC
ZENITH LABS LONGEVITY ACTIVATOR WITH RESVERATROL DOCTOR FORMULATED
ZENWISE HEALTH DAILY DIGESTIVE ENZYMES WITH PREBIOTICS + PROBIOTICS + PLANT-SOURCED BLEND

GREEN TEA
ADVOCARE COREPLEX MULTIPLE VITAMIN AND MINERAL 36 VITAMINS, MINERALS & NUTRIENTS
ADVOCARE MNS 3 METABOLIC NUTRITION SYSTEM ENERGY CONTROL WELLNESS MULTINUTRIENT TRIM
AIM PROANCYNOL 2000 ANTIOXIDANTS WITH GRAPE SEED EXTRACT AIM THE AIM COMPANIES
ANDREW LESSMAN PROCAPS ULTIMATE EYE SUPPORT 12 MG LUTEIN 6 MG ZEAXANTHIN HIGH ANTHOCYANIN BILBERRY AND ELDERBE..
ANDREW LESSMAN PROCAPS ULTIMATE WOMEN'S WELLNESS
ANIMAL PAK THE TRUE ORIGINAL THE ULTIMATE TRAINING PACK
BLUE STAR NUTRACEUTICALS BLADE
BODY FORTRESS SUPER ADVANCED SHRED IGNITER L-LEUCINE L-CARNITINE B-COMPLEX GREEN TEA EXTRACT
BONE & BODY FACTORS CALCIUM-MAGNESIUM INTENSIVE CARE AND MORE PROCAPS LABORATORIES
BOTANIC CHOICE ESSENTIALS GREEN TEA EXTRACT 500 MG.
CRI NATURALS CELLULAR RESEARCH INSTITUTE PROSTALEAF (HERBAL FORMULA)
DEFAULT GREEN TEA
DEFAULT GREENS POWDER
DESIGNS FOR HEALTH FEMGUARD + BALANCE
DOUGLAS COOPER CO. ULTRA OPTICAL CARE 10 MG LUTEIN
ELEMENTAL HEALTH SCIENCES ULTRA-STRENGTH KETOLEAN7 RASPBERRY KETONES CONJUGATED LINOLEIC ACID L-CARNITINE-L-TART..
ESTROVEN MAXIMUM STRENGTH + ENERGY DRUG FREE & ESTROGEN FREE
EVLUTION NUTRITION LEANMODE STIMULANT FREE FAT BURNER
EVLUTION NUTRITION TRANS4ORM ENERGIZED WEIGHT LOSS SUPPORT

© 2024 Likhitsup A et al. *JAMA Network Open*.

GNC MEGA MEN 50 PLUS VITAPAK PROGRAM WITH CLINICALLY STUDIED MULTIVITAMIN

GNC MEGA MEN ENERGY & METABOLISM VITAPAK PROGRAM WITH CLINICALLY STUDIED MULTIVITAMIN BLEND
GNC MULTIVITAMIN ULTRA MEGA GOLD NUTRIENT-DENSE MULTIVITAMIN, MINERAL AND ANTIOXIDANT FORMULA TIMED RELEASE

GNC WOMEN'S HAIR, SKIN & NAILS FORMULA 3,000 MCG OF BIOTIN ANTIOXIDANTS

GNC WOMEN'S ULTRA MEGA ACTIVE CLINICALLY STUDIED MULTIVITAMIN TIMED-RELEASE NEW! SMALLER PILLS
GNC WOMEN'S ULTRA MEGA BONE DENSITY CLINICALLY STUDIED MULTIVITAMIN WITH 2,000 IU OF VITAMIN D-3 1,000 MG OF CAL..

GNC WOMEN'S ULTRA MEGA CLINICALLY STUDIED MULTIVITAMIN TIMED-RELEASE
GREEN FOODS MATCHA GREEN TEA ENERGY BLEND CEREMONIAL GRADE FARM-DIRECT, WHOLE-LEAF AND SHADE-GROWN
GREEN TEA FAT BURNER LIQUID SOFT-GEL CONCENTRATED EXTRACT MAXIMUM STRENGTH 400 MG EGCG HIGHLY CONCENTRATED EGCG

IRWIN NATURALS PROSTA-STRONG SAW PALMETTO PLUS LYCOPENE & PUMPKIN SEED EXTRACT

JOINT FORMULA HEALTHY JOINT FORMULA

KETOLABS CORE BASICS MULTI-VITAMIN-MINERAL
LIFE EXTENSION MEGA GREEN TEA EXTRACT 98% POLYPHENOLS LIGHTLY CAFFEINATED ONE-PER-DAY CONCENTRATED POLYPHENOL EX..
MEGAFOOD MULTI FOR WOMEN 55+ DOCTOR FORMULATED TIERAONA LOW DOG, M.D. WITH BROWN RICE, CARROTS, BROCCOLI & ORANG..
MEGAFOOD MULTI FOR WOMEN 55+ DOCTOR TIERAONA LOW DOG, M.D. FORMULATED WITH BROWN RICE, CARROTS, BROCCOLI & ORANG..

MELALEUCA PROVEXCV
NATURE MADE DAILY DIABETES HEALTH PACK SCIENTIFICALLY FORMULATED FOR PEOPLE WITH PREDIABETES & DIABETES MULTIVIT..

NESTED NATURALS VEGAN OMEGA-3

NEWVITALITY ROYAL GREENS ORIGINAL BLEND
NEWVITALITY RUBY REDS POMEGRANATE, ACAI & MAQUI A DELICIOUS FRUIT AND VEGETABLE WITH POTENT VITAMINS, MINERALS, ..

NORTHSTAR NUTRITIONALS RESTORE FX

NOW CLINICAL STRENGTH PROSTATE HEALTH CLINICAL STRENGTH SAW PALMETTO, BETA-SITOSTEROL & LYCOPENE
NOW EVE SUPERIOR WOMEN'S MULTI SOFTGELS WITH EVENING PRIMROSE, CRANBERRY, GREEN TEA, HORSETAIL SILICA & COQ10
NOW SPORTS MEN'S EXTREME SPORTS MULTI WITH FREE-FORM AMINO ACIDS, ZMA, TRIBULUS, MCT OIL, HERBAL EXTRACTS AND MO..

NUTRACRAFT HAIRXCEL VITAMIN, MINERAL AND HERBAL COMPLEX CONTAINS BIOTIN, FOLIC ACID AND SILICA

NUTRILITE CHOLESTEROL HEALTH CONTAINS GREEN TEA 2 SOFTGELS, 1X A DAY

ORTHO MOLECULAR PRODUCTS MITOCORE

PHYTAGE LABS TINNITUS 911 EAR RINGING RELIEF FORMULA

PRO-COR LABS MULTI FORCE FOR MEN

PURE ENCAPSULATIONS POLYPHENOL NUTRIENTS MULTIVITAMIN/MINERAL FORMULA WITH ADDED POLYPHENOLS
PURITAN'S PRIDE ULTRA MAN MAX DAILY MULTI PREMIUM PERFORMANCE FORMULA FOR MEN HIGH POTENCY TIMED RELEASE

SOURCE NATURALS LIFE FORCE MULTIPLE

SPRING VALLEY DAILY VITAMIN PACK ACTIVE WOMEN PACKETS

SWANSON PREMIUM BRAND GREEN TEA 500 MG

© 2024 Likhitsup A et al. *JAMA Network Open*.

THE HEALTHY LIFE SUPPLEMENTS PAGG DAYTIME POLICOSANOL, ALPHA LIPOIC ACID, GARLIC BULB, GREEN TEA EXTRACT 1200 MG..
THE VITAMIN SHOPPE ULTIMATE MAN HIGH POTENCY MULTIVITAMIN MULTIMINERAL
THE VITAMIN SHOPPE ULTIMATE WOMAN HIGH POTENCY MULTIVITAMIN MULTIMINERAL
TRUNATURE RESVERATROL PLUS 250 MG STANDARDIZED TRANS-RESVERATROL WITH RED WINE EXTRACT, GRAPE SEED EXTRACT, AND ..
ULTALIFE ADVANCED BLOOD PRESSURE SUPPORT CONTAINS GARLIC, HIBISCUS, FORSLEAN & HAWTHORNE BERRY FAST ACTING FORMULA
USANA CELLSENTIALS VITA-ANTIOXIDANT
VIBRANT HEALTH GREEN VIBRANCE PLANT-BASED ADVANCED DAILY SUPERFOOD +25 BILLION PROBIOTICS CLINICALLY FORMULATED ..
VIBRANT HEALTH MAXIMUM VIBRANCE CONTAINS ALL KNOWN NUTRIENTS PLANT-BASED MULTI CLINICALLY FORMULATED VERSION 3..
VIBRANT HEALTH MAXIMUM VIBRANCE CONTAINS ALL KNOWN NUTRIENTS PLANT-BASED MULTI- ADVANCED DAILY FUTUREFOOD CLINIC..
VITALABS LIFE'S VITALITY MULTI-VITAMIN / MINERAL COMPLEX
VITAMIN WORLD ULTRA MAN MAX DAILY MULTI HIGH POTENCY PREMIUM PERFORMANCE FORMULA FOR MEN TIMED RELEASE
WEST MARTIN NUTRIGENOMICS TELOVITE MULTIVITAMIN
ZENITH LABS LONGEVITY ACTIVATOR WITH RESVERATROL DOCTOR FORMULATED

GARCINIA CAMBOGIA
BODYDYNAMIX SLIMVANCE CORE SLIMMING COMPLEX THERMOGENIC
DEFAULT GARCINIA CAMBOGIA
EVLUTION NUTRITION LEANMODE STIMULANT FREE FAT BURNER
GNC MEGA MEN ENERGY & METABOLISM VITAPAK PROGRAM WITH CLINICALLY STUDIED MULTIVITAMIN BLEND
GNC SUPERFOODS APPLE CIDER VINEGAR COMPLETE FORMULA WITH CAYENNE, GINGER AND GARCINIA CAMBOGIA
NUTRAHEALTH GARCINIA CAMBOGIA PLUS ADVANCED NATURAL WEIGHT LOSS FORMULA CONTAINS 95% HCA
NUTRILITE GLUCOSE HEALTH WITH CHROMIUM PICOLINATE 1 CAPSULE, 3X A DAY
ON OPTI-WOMEN HIGH-POTENCY 40+ INGREDIENTS
PARADISE GARCINIA CAMBOGIA 50% HCA
PURELY INSPIRED 100% PURE GARCINIA CAMBOGIA+ WITH GREEN COFFEE
QUALITY ENCAPSULATIONS GARCINIA CAMBOGIA STANDARDIZED TO 60% HCA EXTRA STRONG
SPRING VALLEY GARCINIA CAMBOGIA STANDARDIZED TO 60% HCA
TOP SECRET NUTRITION GARCINIA CAMBOGIA + WHITE KIDNEY BEAN EXTRACT

BLACK COHOSH
DESIGNS FOR HEALTH FEMGUARD + BALANCE
ESTROVEN
ESTROVEN MAXIMUM STRENGTH + ENERGY DRUG FREE & ESTROGEN FREE
ESTROVEN MENOPAUSE RELIEF +MOOD ONCE DAILY CAPLETS
ESTROVEN STRESS PLUS MOOD & MEMORY
NATURE'S BOUNTY BLACK COHOSH 540 MG WHOLE HERB

© 2024 Likhitsup A et al. *JAMA Network Open*.

NATURE'S WAY BLACK COHOSH ROOT 540 MG PER SERVING
REMIFEMIN ESTROGEN-FREE
SPRING VALLEY BLACK COHOSH 40 MG PER SERVING
SWANSON PREMIUM BRAND BLACK COHOSH 540 MG
VITANICA CRAMP BARK EXTRA FORMULATED BY DR. TORI HUDSON

RED YEAST RICE
AMAZING NUTRITION AMAZING FORMULAS RED YEAST RICE 1200 MG PER SERVING
BOTANIC CHOICE RED YEAST RICE PLUS 635 MG.
NATURE'S BOUNTY RED YEAST RICE 600 MG US LAB TESTED PREMIUM FORMULA
NOW RED OMEGA RED YEAST RICE 600 MG WITH COQ10 30 MG PLUS MILK THISTLE & ALPHA LIPOIC ACID
NOW RED OMEGA RED YEAST RICE WITH COQ10 - 30 MG & OMEGA-3 FISH OIL
PIPINGROCK.COM COQ-10 PLUS RED YEAST RICE WITH ALPHA LIPOIC ACID
RED-Q10 A RED YEAST RICE FORMULA
RX VITAMINS NATURLO CHOLESTEROL RED YEAST RICE AND PLANT STEROL BLEND
SPRING VALLEY RED YEAST RICE 600 MG PER CAPSULE
WEIDER RED YEAST RICE PLUS 1200 MG RED YEAST RICE PER 2 TABLETS

ASHWAGANDHA
ALLMAX TESTOFX
BLUE STAR NUTRACEUTICALS GH PEAK
BLUE STAR NUTRACEUTICALS STATUS
DEFAULT ASHWAGANDHA
DOTERRA MITO2MAX ENERGY & STAMINA COMPLEX
EMERITT SERENITY
GAIA HERBS SINGLE HERBS ASHWAGANDHA ROOT
LIFE EXTENSION COGNITEX WITH PREGNENOLONE & BRAIN SHIELD (GASTRODIN)
MEGAFOOD WOMEN'S ONE DAILY MULTIVITAMIN & MINERAL
NATROGIX PRIME MIND BRAIN HEALTH FORMULA
NATURE'S BOUNTY ANXIETY & STRESS RELIEF ASHWAGANDHA KSM-66 CLINICALLY STUDIED INGREDIENTS
NUGENIX MAXX TESTOSTERONE
OLYMPUS LABS SUPERCHARGED PCT KINGS BLOOD
PURE ENCAPSULATIONS ASHWAGANDHA
PURE ESSENCE STRESS SUPPORT SYSTEM
REDCON1 BOOM STICK
RESTORATIVE FORMULATIONS THYROCARE
SCULPTNATION TESTBOOST EXTRA STRENGTH WITH: HORNY GOAT WEED ASHWAGANDHA GINSENG
SIGNAL//OS
TATTVA'S HERBS ASHWAGANDHA MADE WITH CERTIFIED ORGANIC HERBS

© 2024 Likhitsup A et al. *JAMA Network Open*.

VEDIC CARE 100% ASHWAGANDHA POWDER WITHANIA SOMNIFERA PLANT PART USED: LEAF
VITAMIN WORLD ASHWAGANDHA EXTRACT 500 MG STANDARDIZED TO CONTAIN 1.5% WITHANOLIDES STANDARDIZED EXTRACT
VITANICA ADRENAL ASSIST FORMULATED BY DR. TORI HUDSON VEGAN
WEIDER PRIME HEALTHY TESTOSTERONE SUPPORT FOR MEN CLINICALLY TESTED INGREDIENTS ASHWAGANDHA (KSM-66) AND
YOUTHEORY ASHWAGANDHA 1,000 MG
ZENITH LABS LONGEVITY ACTIVATOR WITH RESVERATROL DOCTOR FORMULATED

OTHER BOTANICALS
21ST CENTURY STANDARDIZED HORSE CHESTNUT EXTRACT
365 LIQUID SUPER B12 B COMPLEX ALCOHOL FREE FORMULA 100% DAILY VALUE OR MORE OF SEVEN B VITAMINS
ADVOCARE CRAVE CHECK S.R. VITAMIN AND HERBAL SUSTAINED RELEASE
AKESO MIGRELIEF ORIGINAL FORMULA TRIPLE THERAPY WITH PURACOL
ALFA VITAMINS NATURAL ALFA 369 OMEGA 369 FLAX OIL 1000 MG ORGANIC
AMAZING HERBS BLACK SEED 100% PURE COLD-PRESSED BLACK CUMIN SEED OIL 500 MG CONTAINS MIN. 0.95% THYMOQUINONE 5X-TQ PREMIUM
AMAZING HERBS BLACK SEED 100% PURE COLD-PRESSED BLACK CUMIN SEED OIL CONTAINS MINIMUM OF 0.95% THYMOQUINONE (TQ) 5X-TQ (TQ) THYMOQUINONE MAXIMUM NATURALLY OCCURRING SOURCE
AMERICAN HEALTH ROYAL BRITTANY EVENING PRIMROSE OIL 500 MG 45 MG GLA (9%) 100% PURE SOLVENT FREE
AMERMED MAGNOLIA BARK EXTRACT 4:1
BIO3 SLIM BODY WEIGHT CONTROL TEA CONTAINING ALL-NATURAL HERBS CASSIA, MALLOW AND SAGE.
BODY WISE RIGHT CHOICE PM CHEALTED MINERALS | PHYTONUTRIENTS | ENZYMES
CASSANOVUM ORIGINAL CASSAVA ROOT EXTRACT & FOLIC ACID
CINNAMON 500 MG
COMPLETE ACTIVE MULTIVITAMIN FOR ACTIVE MEN
CRANBERRY 500 MG
CRAZYBULK TESTO-MAX
CTD SPORTS MULTI ELITE WHOLE FOOD MULTIVITAMIN FRUITS & VEGGIES
CVS HEALTH CINNAMON 1000 MG PER SERVING
CVS HEALTH CRANBERRY 4200 MG EQUIVALENT PER SOFTGEL
CVS HEALTH MILK THISTLE 175 MG STANDARDIZED EXTRACT
CVS HEALTH SAW PALMETTO 450 MG WHOLE HERB
DABUR PUDIN HARA PEARLS HERBAL MINT OILS CAPSULES
DEFAULT BARLEY GRASS CAPSULES
DEFAULT CINNAMON
DEFAULT CRANBERRY
DEFAULT CRANBERRY PLUS VITAMIN C
DEFAULT ECHINACEA
DEFAULT ECHINACEA LIQUID
DEFAULT ELDERBERRY
DEFAULT EVENING PRIMROSE OIL
DEFAULT FLAX SEED OIL
DEFAULT GARLIC

© 2024 Likhitsup A et al. *JAMA Network Open*.

DEFAULT GINKGO BILOBA
DEFAULT GINSENG
DEFAULT MACA ROOT
DEFAULT MILK THISTLE
DEFAULT NETTLE
DEFAULT RHODIOLA ROSEA
DEFAULT SAW PALMETTO
DEFAULT TRIBULUS TERRESTRIS POWDER
DEFAULT VALERIAN ROOT
DH DESERT HARVEST SUPER-STRENGTH FREEZE-DRIED ALOE VERA
DIETWORKS GREEN COFFEE BEAN EXTRACT STANDARDIZED TO 45% CHLOROGENIC ACID WEIGHT MANAGEMENT FORMULA MADE WITH SVETOL
DR. DAVID WILLIAMS PROBIOTIC ADVANTAGE COLON HEALTH EXTRA STRENGTH 10 BILLION LIVE PROBIOTICS
ECLECTIC INSTITUTE RAW FRESH FREEZE-DRIED NETTLE ROOT 300 MG
EMERALD EUROPEAN GINKGO BILOBA 24% GINKGOFLAVONOGLYCOSIDES 6% TERPENE LACTONES
EMSQUARED HOPE IMMUNALOE ALOE VERA ACEMANNAN
EQUAZEN FAMILY TRIPLE STRENGTH EPA DHA GLA HIGHLY CONCENTRATED NATURALLY SOURCED OMEGA-3 WITH OMEGA-6 5 YEARS TO ADULT ONE-A-DAY CAPSULES
ESSENTIAL BEING GINSENG 250 MG
FINEST NUTRITION CINNAMON 1000 MG PER TWO CAPSULES
FINEST NUTRITION CRANBERRY PLUS VITAMIN C 4200 MG PER SOFTGEL
FINEST NUTRITION ECHINACEA 400 MG PER CAPSULE
FINEST NUTRITION ODORLESS GARLIC 1000 MG PER SOFTGEL
FOREVER GARLIC-THYME ODORLESS GARLIC PLUS THYME
FRESH HEALTHCARE PURE PREMIUM MORINGA 1000 MG PER SERVING HIGH STRENGTH COMPLETE GREEN SUPERFOOD RICH SOURCE OF PHYTONUTRIENTS CONTAINS ESSENTIAL AMINO ACIDS SUITABLE FOR VEGETARIANS
GARDEN OF LIFE MYKIND ORGANICS EXTRA STRENGTH TURMERIC WITH ORGANIC FERMENTED WHOLE TURMERIC & GINGER ORGANIC BLACK PEPPER & PROBIOTICS
GARDEN OF LIFE VITAMIN CODE RAW ONE FOR WOMEN WHOLE FOOD
GAT SPORT TESTROL ORIGINAL ORIGINAL FORMULA
GNC HERBAL PLUS ASTRAGALUS 500 MG WHOLE HERB
GNC HERBAL PLUS CAT'S CLAW BARK 500 MG WHOLE HERB
GNC HERBAL PLUS ECHINACEA & GOLDENSEAL VEGETARIAN STANDARDIZED EXTRACT BLEND
GNC HERBAL PLUS HORSE CHESTNUT EXTRACT 300 MG VEGETARIAN STANDARDIZED EXTRACT
GNC HERBAL PLUS MACA ROOT 525 MG VEGETARIAN WHOLE HERB
GNC MILK THISTLE STANDARDIZED HERB EXTRACT VEGETARIAN 200MG
GNC PYCNOGENOL 50 MG
GREENLEAF OPTIMUM HEALTH CHOLESTEROL SUPPORT
HAMPSHIRE LABS, INC. THE ULTIMATE DIABETIC CIRCULATION BOOSTER
HAVASU NUTRITION L-ARGININE EXTRA STRENGTH
HELIOCARE CLINICALLY PROVEN FERNBLOCK PLE TECHNOLOGY
HERBALFACTORS RHODIOLA STANDARIZED TO 3% SALIDROSIDES 150 MG NATURAL FACTORS
HERBALIFE CELL ACTIVATOR FORMULA 3
IBGARD INDIVIDUALLY TRIPLE-COATED, SUSTAINED-RELEASE MICROSPHERES OF ULTRAMEN, AN ULTRAPURIFIED PEPPERMINT OIL

© 2024 Likhitsup A et al. *JAMA Network Open*.

IRON ADDICTS BRAND SLEEVE BUSTER PUMP FORMULA
IT WORKS! ADVANCED FORMULA FATFIGHTER WITH CARB INHIBITORS
JARROW FORMULAS ARTICHOKE STANDARDIZED EXTRACT 500 MILLIGRAMS
KIRKLAND SIGNATURE HAIR, SKIN & NAILS HIGH POTENCY BIOTIN 5000 MCG PLUS ANTIOXIDANTS VITAMIN A & C - CALCIUM - VITAMIN D3 WITH KERATIN
KYOLIC AGED GARLIC EXTRACT ORIGINAL FORMULA FORMULA 100 ODORLESS ORGANIC GARLIC
LIFE EXTENSION BLUEBERRY EXTRACT WITH POMEGRANATE FULL-SPECTRUM FOOD-BASED POLYPHENOLS
LIFE EXTENSION OMEGA FOUNDATIONS SUPER OMEGA-3 EPA/DHA WITH SESAME LIGNANS & OLIVE EXTRACT SMALL, EASY-TO-SWALLOW SOFTGELS
LITTLE REMEDIES FAST ACTING GRIPE WATER NEWBORN+
MAX EFFORT MUSCLE ME PRE WORKOUT
MAXIMUM STRENGTH VIGOR THRIVE MALE ENHANCEMENT COMPLEX NEW IMPROVED FORMULA
MAXIVISION EYE VITAMIN & MULTIVITAMIN WHOLE BODY FORMULA AREDS2 BASED 2X LUTEIN & ZEAXANTHIN 20 MG LUTEIN + 4 MG ZEAXANTHIN PLUS PROPRIETARY BLEND
MEGAFOOD BALANCED B COMPLEX WITH KALE, BROCCOLI & BROWN RICE MULTIVITAMIN FOXY ORGANIC
MEGAFOOD BLOOD BUILDER MINIS WITH BEETS, ORANGES & BROCCOLI IRON & MULTIVITAMIN
MEGAFOOD MEGAFLORA FOR WOMEN 50 BILLION ACTIVE CULTURES WITH CRANBERRY & PREBIOTIC FOODS PROBIOTIC
MEMBER'S MARK CLINICAL STRENGTH CRANBERRY CAPSULES, 500 MG MADE WITH 100% NATURAL WHOLE CRANBERRIES
MEMBER'S MARK EASY TO SWALLOW VITAMIN C 1000 MG WITH ZINC NATURAL ROSE HIPS AND CITRUS BIOFLAVONOIDS
MEMBER'S MARK HIGH ABSORPTION TURMERIC CURCUMIN COMPLEX WITH STANDARDIZED EXTRACT CAPSULES, 500 MG VEGETARIAN CAPSULE
MEMBER'S MARK STANDARDIZED EXTRACT GINKGO BILOBA SOFTGELS, 120 MG
MEMBER'S MARK VITAMIN C WITH NATURAL ROSE HIPS TABLETS, 1000 MG
MORTAR AND PESTLE HERBS OIL OF OREGANO MADE WITH ORGANIC OREGANO
NATROL B-COMPLEX FAST DISSOLVE CONTAINS ALL B VITAMINS VEGETARIAN
NATURAL MENTE BIO-CELL STEM-CELL COMPLEX ALL NATURAL
NATURE MADE FLAXSEED OIL MADE WITH ORGANIC FLAXSEED OIL 1000 MG
NATURE MADE WHOLE HERB ECHINACEA (PURPUREA) 350 MG AERIAL PART
NATURE'S BLEND ECHINACEA 350 MG AND GOLDEN SEAL 100 MG
NATURE'S BOUNTY CINNAMON 1000 MG PER SERVING
NATURE'S BOUNTY CRANBERRY FRUIT 4200 MG EQUIVALENT PER SOFTGEL | WITH VITAMIN C
NATURE'S BOUNTY CRANBERRY FRUIT CONCENTRATE 25,200 MG EQUIVALENT PER SERVING WITH VITAMIN C
NATURE'S BOUNTY DOUBLE STRENGTH STANDARDIZED EXTRACT GINKGO BILOBA 120 MG STANDARDIZED TO CONTAIN 24% GINKGO FLAVONE GLYCOSIDES
NATURE'S BOUNTY ECHINACEA 400 MG | WHOLE HERB
NATURE'S BOUNTY FLAXSEED OIL 1200 MG | 540 MG OF OMEGA-3
NATURE'S BOUNTY GARLIC 2000 MG EQUIVALENT PER TABLET
NATURE'S BOUNTY GARLIC EXTRACT 1000 MG EQUIVALENT
NATURE'S BOUNTY GINGER ROOT 550 MG | WHOLE HERB
NATURE'S BOUNTY GINKGO BILOBA 60 MG PER SERVING | STANDARDIZED TO CONTAIN 24% GINKGO FLAVONE GLYCOSIDES
NATURE'S BOUNTY GINSENG COMPLEX
NATURE'S BOUNTY MILK THISTLE 175 MG | STANDARDIZED EXTRACT
NATURE'S BOUNTY ODORLESS GARLIC AND PARSLEY
NATURE'S BOUNTY SAW PALMETTO 450 MG | WHOLE HERB

© 2024 Likhitsup A et al. *JAMA Network Open*.

NATURE'S LIFE ALFALFA LEAF 1,000 MG MEDICAGO SATIVA 2 DAILY
NATURE'S MEASURE GINKGO BILOBA 4:1 EXTRACT
NATURE'S MEASURE GINSENG 250 MG
NATURE'S MEASURE ODOR CONTROLLED GARLIC 1000 MG
NATURE'S PLUS HEMA-PLEX CAPSULES IRON WITH SYNERGISTIC COFACTORS 85 MG ELEMENTAL IRON MILD AND GENTLE FAST-ACTING VEGAN
NATURE'S PLUS HERBAL ACTIVES NUTRIZAC 300 MG ST. JOHN'S WORT STANDARDIZED BOTANICAL NOW WITH METHYLCOBALAMIN!
NATURE'S PLUS SOURCE OF LIFE MULTI-VITAMIN & MINERAL WITH WHOLE FOOD CONCENTRATES TABLETS VEGETARIAN
NATURE'S SECRET 15-DAY WEIGHT LOSS SUPPORT CLEANSE & FLUSH
NATURE'S SUNSHINE GARLIC
NATURE'S SUNSHINE HAWTHORN BERRIES
NATURE'S SUNSHINE SAW PALMETTO
NATURE'S SUNSHINE ST. JOHN'S WORT CONCENTRATED
NATURE'S TRUTH VITAMINS GINKGO BILOBA STANDARDIZED EXTRACT 120 MG PLUS BACOPA EXTRACT
NATURE'S TRUTH VITAMINS TURMERIC CURCUMIN COMPLEX 500 MG PLUS BLACK PEPPER EXTRACT
NATURE'S WAY FORTIFY AGE 50+ PROBIOTIC +PREBIOTICS EVERYDAY CARE 30 BILLION LIVE PROBIOTIC CULTURES PER CAPSULE 11 PROBIOTIC STRAINS PROBIOTIC
NATURE'S WAY HYSSOP HERB 900 MG PER SERVING
NATURE'S WAY PREMIUM EXTRACT ASTRAGALUS 0.5% ASTRAGALOSIDES
NATURE'S WAY SARSAPARILLA ROOT 425 MG
NATURE'S WAY STANDARDIZED GRAPE SEED 95% POLYPHENOLS
NATURE'S WAY STANDARDIZED MILK THISTLE 80% SILYMARIN
NATURE'S WAY STANDARDIZED OLIVE LEAF 20% OLEUROPEIN
NATURE'S WAY STANDARDIZED OREGANO OIL 75-85% CARVACROL
NATURE'S WAY THISILYN STANDARDIZED MILK THISTLE EXTRACT MAXIMUM ABSORPTION LIVER SUPPORT FORMULA STANDARDIZED TO 80% SILYMARIN VEGETARIAN
NATURE'S WAY VITEX FRUIT 400 MG
NATURELO ONE DAILY MULTIVITAMIN FOR WOMEN
NOW ADAM SOFTGELS SUPERIOR MEN'S MULTI WITH SAW PALMETTO, PLANT STEROLS, LYCOPENE & COQ10
NOW GARLIC OIL 1500 MG EQUIVALENT TO WHOLE CLOVE GARLIC
NOW GINGER ROOT 550 MG VEGETARIAN/VEGAN
NOW HORNY GOAT WEED EXTRACT 750 MG SUITABLE FOR MEN AND WOMEN PLUS 150 MG OF MACA ROOT VEGETARIAN/VEGAN NON-GMO
NOW OLIVE LEAF EXTRACT 500 MG STANDARDIZED 6% OLEUROPEIN
NOW SAW PALMETTO EXTRACT WITH PUMPKIN SEED OIL AND ZINC
NOW SPORTS TRIBULUS 1,000 MG STANDARDIZED EXTRACT MINIMUM 45% SAPONINS VEGETARIAN/VEGAN NON-GMO
NOW VITAMIN K-2 100 MCG BIOLOGICALLY ACTIVE FORM MENAQUINONE-4 (MK-4) VEGETARIAN/VEGAN NON-GMO
NUGENIX TESTOSTERONE MULTIVITAMIN MEN'S DAILY TESTOSTERONE MULTIVITAMIN
NUSAPURE FRENCH MARITIME PINE BARK EXTRACT 300 MG PER SERVING
NUTRIBIOTIC VEGAN GSE GRAPEFRUIT SEED EXTRACT LIQUID CONCENTRATE UNFILTERED FORMULA!
NUTRICOST BITTER MELON MOMORDICA CHARANTIA 600MG EXTRACT PER SERVING
NUTRIFLAIR CEYLON CINNAMON MADE WITH ORGANIC CEYLON CINNAMON 1200 MG PER SERVING
NUTRILITE CLEARGUARD 2 TABLETS, 3X A DAY
NUTRILITE COMPLEX FOR HAIR, SKIN AND NAILS

© 2024 Likhitsup A et al. *JAMA Network Open*.

NUTRILITE DOUBLE X 22 VITAMINS AND MINERALS AND 20 PLANT CONCENTRATES 3 TABLETS, 2X A DAY
NUTRILITE HAIR, SKIN & NAIL HEALTH CONTAINS BIOTIN AND COLLAGEN 1 TABLET, 1X A DAY
NUTRILITE HEART HEALTH COQ10 1-3 SOFTGELS, 1X A DAY
NUTRILITE IRON FOLIC 1 TABLET, 1-3X A DAY
NUTRILITE JOINT HEALTH GLUCOSAMINE AND CHONDROITIN 2 TABLETS, 2X A DAY
NUTRILITE MEMORY BUILDER CISTANCHE 2 TABLETS, 1X A DAY
NUTRILITE MEN'S PACK CONVENIENT DAILY VITAMIN AND MINERAL NUTRITION FOR MEN 1 PACKET A DAY
NUTRILITE PERFECT PACK WITH 22 VITAMINS AND MINERALS, AND 22 PLANT CONCENTRATES INCLUDES DOUBLE X 2 PACKETS A DAY
NUTRILITE PROSTATE HEALTH NATURAL SUPPORT WITH SAW PALMETTO AND NETTLE ROOT 1 SOFTGEL, 3X A DAY
NUTRILITE VITAMIN B DUAL-ACTION B VITAMINS 1 TABLET, 1X A DAY
NUTRILITE WOMEN'S PACK CONVENIENT DAILY VITAMIN AND MINERAL NUTRITION FOR WOMEN 1 PACKET A DAY
OREGON'S WILD HARVEST CHASTE TREE MADE WITH ORGANIC CHASTE TREE
OREGON'S WILD HARVEST KELP NATURAL SOURCE OF IODINE
ORGANIC INDIA NEEM CERTIFIED ORGANIC
OXYLENT DRINK OXYLENT BREATHE LIFE 5-IN-1 MULTIVITAMIN DRINK DAILY ESSENTIAL NUTRIENTS PACKETS
PHARMA SERIES BETALEAN-SCA ANSI
PLEXUS PROBIO 5
PRIMAL LABS CARDIORELAX AO
PROSA NOPAL (OPUNTIA FICUS INDICA) 400 MG
PURITAN'S PRIDE BILBERRY EXTRACT 60 MG STANDARDIZED TO CONTAIN 25% ANTHOCYANOSIDES STANDARDIZED EXTRACT
PURITAN'S PRIDE EXTRA STRENGTH PROSTA-METTO SAW PALMETTO COMPLEX STANDARDIZED EXTRACT FLOW GUARD
PURITAN'S PRIDE GRAPESEED EXTRACT 100 MG STANDARDIZED FOR POLYPHENOLS
PURITAN'S PRIDE NATURAL FLAX OIL 1000 MG OMEGA-3, 6 & 9 COLD PRESSED
PURITAN'S PRIDE PREMIUM ECHINACEA 400 MG NATURAL WHOLE HERB
PURITAN'S PRIDE PREMIUM ODORLESS GARLIC 500 MG GARLIC FRESH
PURITAN'S PRIDE SAW PALMETTO STANDARDIZED TO CONTAIN 85-95% FATTY ACIDS & STEROLS 160 MG STANDARDIZED EXTRACT
PURITY PRODUCTS ASTAFX ASTAREAL ASTAXANTHIN MIXED TOCOTRIENOLS BIOPERINE
PURITY PRODUCTS B-12 ENERGY BERRY LEMONADE MELT WITH VITAMIN B6, FOLIC ACID, VITAMIN D
PURITY PRODUCTS B-12 ENERGY BERRY MELT WITH VITAMIN B6, FOLIC ACID, VITAMIN D
PURITY PRODUCTS EVERSTRONG CREAPURE CREATINE COFFEEBERRY - VITAMIN D FRUITEX-B PHYTOBORON
QUANTUM HEALTH SUPER LYSINE+ WITH VITAMIN C, ECHINACEA, LICORICE, PROPOLIS, ODORLESS GARLIC
REAL HEALTH THE PROSTATE FORMULA WITH SAW PALMETTO ADVANCED FORMULATION NOW WITH VITAMIN D & GINGER!
RENEW LIFE WOMEN'S DAILY 2-IN-1 PREBIOTICS + PROBIOTICS 20 BILLION LIVE CULTURES 10 PROBIOTIC STRAINS PROBIOTIC
REXALL CONCENTRATED CRANBERRY FRUIT 4200 MG EQUIVALENT PER SOFTGEL PLUS VITAMIN C
REXALL ELDERBERRY SAMBUCUS 1250 MG EQUIVALENT JUICE CONCENTRATE
REXALL NATURALIST ODORLESS GARLIC EXTRACT 1000 MG EQUIVALENT PER SOFTGEL
REXALL ODORLESS GARLIC 500 MG
REXALL STANDARDIZED EXTRACT GINKGO BILOBA 120 MG
REXALL STANDARDIZED EXTRACT KOREAN GINSENG 100 MG
RITE AID PHARMACY ECHINACEA 380 MG

© 2024 Likhitsup A et al. *JAMA Network Open*.

SANAR NATURALS COLLAGEN WRINKLE FORMULA
SAW PALMETTO 160 MG EUROPEAN STANDARDIZED STANDARDIZED TO 85%-95% [136-152 MG PER CAPSULE] FATTY ACIDS AND STEROLS
SHAKLEE +GREENS ALFALFA COMPLEX ULTRA-PURE, PREMIUM ALFALFA & SPEARMINT
SHOT-B GINSENG 40.0 MULTIVITAMIN AND GINSENG EXTRACT
SIMPLY RIGHT NATURAL ROSE HIPS VITAMIN C 1000 MG HIGH POTENCY WITH BIOFLAVONOIDS
SMOKY MOUNTAIN NATURALS DIM 200 MG + BIOPERINE EXTRA STRENGTH FORMULA
SOLARAY BAMBOO STEM EXTRACT 300 MG PER CAPSULE BAMBUSA VULGARIS 70% SILICA
SOLARAY DEVIL'S CLAW HARPAGOPHYTUM SPP. 525 MG PER CAPSULE WHOLE ROOT
SOLARAY GARLIC ALLIUM SATIVUM 500 MG PER CAPSULE
SOLARAY MANGOSTEEN GARCINIA MANGOSTANA 475 MG PER CAPSULE WHOLE FRUIT
SOLARAY PASSION FLOWER 350 MG PER CAPSULE WHOLE AERIAL PASSIFLORA INCARNATA
SOLARAY TWO-STAGE, TIMED-RELEASE SUPER BIO C BUFFERED FAST-ACTING FIRST STAGE TIMED-RELEASE SECOND STAGE
SOLARAY VITEX 400 MG PER CAPSULE WHOLE BERRY VITEX ANGUS-CASTUS
SOURCE NATURALS WELLNESS FORMULA HERBAL DEFENSE COMPLEX TABLETS
SPRING VALLEY BILBERRY EXTRACT 150 MG PER CAPSULE MADE WITH 150 MG ORGANIC BILBERRY EXTRACT, 100 MG ORGANIC BLUEBERRY EXTRACT, AND 6 MG ORGANIC LUTEIN WITH BILBERRY, LUTEIN AND BLUEBERRY
SPRING VALLEY C 500 MG WITH ROSE HIPS
SPRING VALLEY CINNAMON 1000 MG PER SERVING 2 PER DAY
SPRING VALLEY CINNAMON 500 MG PER CAPSULE PLUS CHROMIUM
SPRING VALLEY COLD-PRESSED FLAXSEED OIL 1000 MG PER SOFTGEL 450 MG OMEGA-3
SPRING VALLEY CONCENTRATED ALOE VERA GEL
SPRING VALLEY CONCENTRATED EXTRACT ACAI
SPRING VALLEY CONCENTRATED ODOR-CONTROLLED GARLIC 1,000 MG PER SOFTGEL
SPRING VALLEY CONCENTRATED ODOR-CONTROLLED GARLIC 1000 MG
SPRING VALLEY ECHINACEA
SPRING VALLEY ECHINACEA 760 MG PER SERVING MADE WITH ORGANIC ECHINACEA POWDER
SPRING VALLEY EXTRA STRENGTH HAIR, SKIN & NAILS 5000 MCG BIOTIN PER SERVING WITH ARGAN OIL, COCONUT OIL, AND COLLAGEN
SPRING VALLEY FENUGREEK 610 MG PER CAPSULE MADE WITH ORGANIC FENUGREEK SEED POWDER
SPRING VALLEY GINKGO BILOBA EXTRACT 120 MG PER CAPSULE
SPRING VALLEY HAWTHORN BERRIES 565 MG PER CAPSULE MADE WITH ORGANIC HAWTHORN BERRY POWDER
SPRING VALLEY STANDARDIZED EXTRACT CRANBERRY 84 MG PER SOFTGEL
SPRING VALLEY STANDARDIZED EXTRACT GINKGO BILOBA 60 MG PER TABLET
SPRING VALLEY STANDARDIZED EXTRACT KOREAN PANAX GINSENG 100 MG PER CAPSULE
SPRING VALLEY STANDARDIZED EXTRACT MILK THISTLE 175 MG PER CAPSULE
SPRING VALLEY STANDARDIZED EXTRACT SAW PALMETTO 160 MG PER SOFTGEL
SPRING VALLEY STANDARDIZED EXTRACT ST. JOHN'S WORT 150 MG PER CAPSULE
SPRING VALLEY TART CHERRY EXTRACT 1200 MG PER CAPSULE MADE WITH ORGANIC TART CHERRY EXTRACT
SPRING VALLEY ULTRA TRIPLE STRENGTH CRANBERRY 15,000 MG EQUIVALENT PER CAPSULE
SPRING VALLEY VALERIAN ROOT 500 MG PER SERVING MADE WITH ORGANIC VALERIAN ROOT
SPRING VALLEY WHOLE HERB GINGER ROOT 550 MG PER CAPSULE
SPRING VALLEY WHOLE HERB SAW PALMETTO 450 MG PER CAPSULE
SPRING VALLEY WOMEN'S PROBIOTIC 1 BILLION ACTIVE CULTURES PER CAPSULE WITH CRANBERRY EXTRACT

© 2024 Likhitsup A et al. *JAMA Network Open*.

SPROUTS FARMERS MARKET 100% VEGETARIAN OREGANO OIL CARVACROL 70
SPROUTS FARMERS MARKET ST. JOHN'S WORT
SUNDOWN NATURALS CINNAMON 1000 MG PER SERVING
SUNDOWN NATURALS STANDARDIZED MILK THISTLE 240 MG PER SERVING
SUNDOWN NATURALS WHOLE HERB SAW PALMETTO 450 MG
SUPERIOR SOURCE NO SHOT CYANOCOBALAMIN B-12 1000 MCG B-6 & FOLIC ACID 400 MCG
SWANSON GRAPE SEED EXTRACT 200 MG PER CAPSULE
SWANSON PREMIUM BRAND PSYLLIUM HUSKS 610 MG
SWANSON PROBIOTICS PRUNE REGULARITY COMPLEX WITH PROBIOTICS & FOS 5 BILLION CFU
SWANSON ULTRA JOINT CARE WITH GLUCOSAMINE, MSM & CHONDROITIN
SWANSON ULTRA SUPER STRENGTH CRANBERRY WHOLE FRUIT CONCENTRATE
TERRY NATURALLY SAGAPRO BLADDER HEALTH FOR MEN AND WOMEN ANGELICA CLINICALLY STUDIED
TESTMAX CYCLE 45 DOUBLE DOSE FORMULA FOR MEN
TESTOGEN
TESTOMENIX
THE PEOPLE'S CHEMIST IMMUNE FX
THE VITAMIN SHOPPE CINNAMON EXTRACT 500 MG
THE VITAMIN SHOPPE CRANBERRY ACTION + VITAMIN C
THE VITAMIN SHOPPE ECHINACEA ECHINACEA PURPUREA 400 MG HERB
THE VITAMIN SHOPPE FENUGREEK SEED A TRADITIONAL HERB 610 MG
THE VITAMIN SHOPPE MILK THISTLE EXTRACT 70 MG 80% SILYMARIN
THERALOGIX THERACRAN ONE CRANBERRY STANDARDIZED TO CONTAIN 36 MG OF CRANBERRY PACS PER CAPSULE
TOTAL CHELATE NUTRI-WEST
TRADER JOE'S B-6 / FOLIC ACID / B-12 UNDER THE TONGUE HIGH POTENCY
TRADER JOE'S SYNERGISTIC C VITAMIN C COMPLEX 500 MG PER TABLET WITH LEMON BIOFLAVONOID COMPLEX AND RUTIN BIOFLAVONOIDS
TRADITIONAL MEDICINALS ORGANIC ECHINACEA PLUS ORIGINAL WITH SPEARMINT CAFFEINE FREE
TRADITIONAL MEDICINALS ORGANIC THROAT COAT ORIGINAL WITH SLIPPERY ELM CAFFEINE FREE
TRUNATURE ADVANCED STRENGTH CINSULIN WATER EXTRACT OF CINNAMON CINNAMON CONCENTRATE 10:1 STRENGTH 2 CAPSULES DELIVER 500 MG CINNAMON EXTRACT PLUS 400 MCG CHROMIUM AND 12.5 MCG (500 IU) VITAMIN D
TRUNATURE ADVANCED STRENGTH CINSULIN WATER EXTRACT OF CINNAMON CINNAMON CONCENTRATE 10:1 STRENGTH 2 CAPSULES DELIVER 500 MG CINNAMON EXTRACT PLUS 400 MCG CHROMIUM AND 500 IU VITAMIN D3 VEGGIE CAPS!
TRUNATURE CRANBERRY ANTIOXIDANT ACTIVITY WITH SHANSTAR CONCENTRATED EXTRACT 300 MG
TRUNATURE GINKGO BILOBA 120 MG WITH VINPOCETINE STANDARDIZED HERBAL EXTRACT
TRUNATURE GRAPE SEED & RESVERATROL ONE PER DAY TIMED-RELEASE FOR SUSTAINED ACTION PLUS BENEFITS OF WHOLE GRAPE WITH RESVERATROL 75 MG
TRUNATURE PROSTATE PLUS HEALTH COMPLEX STANDARDIZED SAW PALMETTO WITH ZINC, LYCOPENE & PUMPKIN SEED CRAN-MAX CRANBERRY JUST 1 SOFTGEL PER DAY WITH CRAN-MAX CRANBERRY
UNICITY ALOE VERA
UNICITY RENEW FOR MEN
VESELE
VH ESSENTIALS PROBIOTICS WITH PREBIOTICS & CRANBERRY
VITAMIN WORLD MORINGA MORINGA OLEIFERA 5000 MG CONCENTRATED HERB
VITAMIN WORLD SAW PALMETTO 1000 MG 4:1 EXTRACT CONCENTRATED HERB

© 2024 Likhitsup A et al. *JAMA Network Open*.

VYANTIXRX MALE ENHANCEMENT FORMULA
WALGREENS HAIR, SKIN AND NAILS FORMULA WITH BIOTIN & KERATIN TWO TABLETS PER DAY
WINK NATURALS PRE & PRO BIOTIC HUCKLEBERRY
YOUNG LIVING ESSENTIAL OILS PROSTATE HEALTH ESSENTIAL OILS

© 2024 Likhitsup A et al. *JAMA Network Open*.

eFigure. Herbal and Dietary Supplement Use Among U.S. Adults Enrolled in NHANES 2017-2020

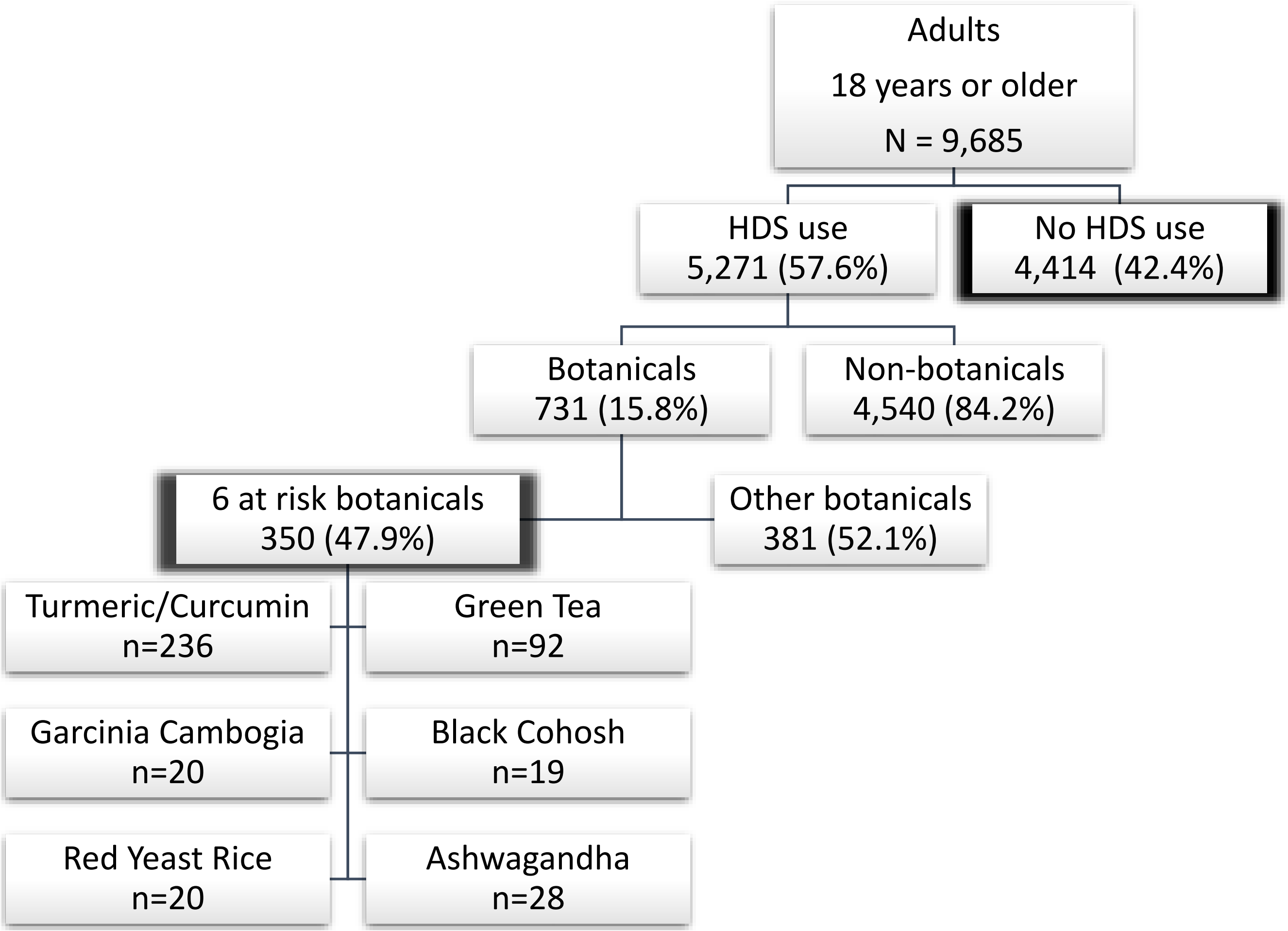

eFigure 1 legend: Amongst 9,685 adults enrolled in NHANES 2017-2020 cohort, 57.6% reported using at least 1 HDS product within the past 30 days. Amongst the 731 adults using botanicals, 350 participants (4.7%) used at least one of the 6 at risk botanical products

© 2024 Likhitsup A et al. *JAMA Network Open*.
