# Supplementary material for: Estimated Exposure to 6 Potentially Hepatotoxic Botanicals in US Adults
Source: JAMA Netw Open. 2024 Aug 5;7(8):e2425822. doi: 10.1001/jamanetworkopen.2024.25822 (PMC11301549; doi:10.1001/jamanetworkopen.2024.25822)
Supplement: Supplement 2. — Data Sharing Statement [file jamanetwopen-e2425822-s002.pdf]

Data Sharing Statement

Likhitsup. Estimated Exposure to 6 Potentially Hepatotoxic Botanicals in US Adults. *JAMA Netw Open*. Published August 05, 2024. doi:10.1001/jamanetworkopen.2024.25822

Data
Data available: No

Additional Information
Explanation for why data not available: NHANES data is available publicly via CDC website
